# Transcriptome Landscape of Epithelial to Mesenchymal Transition of Human Stem Cell–Derived RPE

**DOI:** 10.1167/iovs.62.4.1

**Published:** 2021-04-01

**Authors:** Srinivasa R. Sripathi, Ming-Wen Hu, Melissa M. Liu, Jun Wan, Jie Cheng, Yukan Duan, Joseph L. Mertz, Karl J. Wahlin, Julien Maruotti, Cynthia A. Berlinicke, Jiang Qian, Donald J. Zack

**Affiliations:** 1 Department of Ophthalmology, Stem Cell Ocular Regenerative Medicine Center, Wilmer Eye Institute, The Johns Hopkins University School of Medicine Baltimore, Maryland, United States; 2 Department of Medical and Molecular Genetics, Indiana University School of Medicine, Indianapolis, Indiana, United States; 3 Shiley Eye Institute, University of California, San Diego, LA Jolla, California, United States; 4 Phenocell, Evrycedex, France; 5 Solomon H. Snyder Department of Neuroscience, Department of Molecular Biology and Genetics, Department of Genetic Medicine, Center for Nanomedicine at the Wilmer Eye Institute, The Johns Hopkins University School of Medicine, Baltimore, Maryland, United States

**Keywords:** stem cells, differentiation, retinal pigment epithelium, epithelial–mesenchymal transition, TGF-β/TNF-α, transcriptomics, PVR, AMD

## Abstract

**Purpose:**

RPE injury often induces epithelial to mesenchymal transition (EMT). Although RPE-EMT has been implicated in a variety of retinal diseases, including proliferative vitroretinopathy, neovascular and atrophic AMD, and diabetic retinopathy, it is not well-understood at the molecular level. To contribute to our understanding of EMT in human RPE, we performed a time-course transcriptomic analysis of human stem cell-derived RPE (hRPE) monolayers induced to undergo EMT using 2 independent, yet complementary, model systems.

**Methods:**

EMT of human stem cell-derived RPE monolayers was induced by either enzymatic dissociation or modulation of TGF-β signaling. Transcriptomic analysis of cells at different stages of EMT was performed by RNA-sequencing, and select findings were confirmed by reverse transcription quantitative PCR and immunostaining. An ingenuity pathway analysis (IPA) was performed to identify signaling pathways and regulatory networks associated with EMT.

**Results:**

Proteocollagenolytic enzymatic dissociation and cotreatment with TGF-β and TNF-α both induce EMT in human stem cell-derived RPE monolayers, leading to an increased expression of mesenchymal factors and a decreased expression of RPE differentiation-associated factors. Ingenuity pathway analysis identified the upstream regulators of the RPE-EMT regulatory networks and identified master switches and nodes during RPE-EMT. Of particular interest was the identification of widespread dysregulation of axon guidance molecules during RPE-EMT progression.

**Conclusions:**

The temporal transcriptome profiles described here provide a comprehensive resource of the dynamic signaling events and the associated biological pathways that underlie RPE-EMT onset. The pathways defined by these studies may help to identify targets for the development of novel therapeutic targets for the treatment of retinal disease.

Epithelial to mesenchymal transition (EMT) produces mesenchymal cells for development and tissue repair, but it can also contribute to tissue pathology.^1^^,^^2^ EMT is characterized by the loss of epithelial cell characteristics, such as cell polarity and cellular adhesiveness, and the acquisition of a mesenchymal cell-like characteristics, such as increased cellular motility and invasiveness. During early EMT, changes in gene expression profiles correlate with the reorganization of the cytoskeletal architecture, resulting in a loss of integral tight junctions, as well as a disrupted apicobasal polarity and cell morphology.^3^^,^^4^ Most EMT studies to date have focused on embryonic development, tissue fibrosis, and tumor metastasis.^2^^,^^5^^,^^6^ However, more recently there has been increasing interest in the role of EMT in ocular disease, particularly EMT affecting the RPE, given the increasing data implicating RPE EMT in the pathogenesis of proliferative vitroretinopathy,^7^^–^^10^ neovascular (“wet”) AMD,^11^^,^^12^ atrophic (“dry”) AMD,^13^^,^^14^ and diabetic retinopathy.^15^

Developments in stem cell biology now allow the differentiation of human embryonic stem cells and induced pluripotent stem cells into RPE cells (human stem cell-derived RPE [hRPE]) that closely mimic the morphologic, biochemical, molecular, and functional characteristics of native RPE.^16^^–^^18^Additionally, the availability of these cells is making possible the development of model systems to examine the key factors and mechanisms involved in RPE-EMT. Based on the finding that growth factor TGF-β and cytokine TNF-α induce EMT onset in embryonic development, tumor progression, and tissue fibrosis,^19^^,^^20^ Boles et al.^10^ used TGF-β/TNF-α to induce and study EMT in hRPE cells. We developed a similar model system for studying hRPE EMT and found that cotreatment with TGF-β/TNF-α induced differential regulation of several mesenchymal and epithelial-specific transcription factors, consistent with classically defined EMT onset. We found that enzymatic dissociation of hRPE monolayers also recapitulated many aspects of the cancer-associated EMT transcription factor expression profile, providing a second in vitro RPE-EMT model. Here we present and contrast the transcriptional changes of hRPE monolays subject to EMT induced by either treatment with TGF-β/TNF-α or by enzymatic dissociation.

## Methods

### Human Pluripotent Stem Cell Culture and Differentiation into RPE

For the studies described here we used the EP1^21^ and induced pluripotent stem cells (IMR90)-4 (WiCell, Madison, WI) human pluripotent stem cell lines. The human pluripotent stem cells were cultured and differentiated into RPE monolayers as described previously.^16^^,^^17^

### Flow Cytometry Analysis

Immunostaining for RPE-specific markers were performed using the IntraPrep Permeabilization kit (Beckman Coulter, Brea, CA) as per the manufacturer's instructions. Primary antibody concentration was 1 μg/1 million cells for mouse anti-PMEL17 (Abcam, Cambridge, MA), mouse anti-RPE65 (Abcam). Goat anti-mouse conjugated to Alexa 647 (Invitrogen, Carlsbad, CA) was used as a secondary antibody. Nonspecific, species-appropriate isotype control was included in all flow cytometry experiments and stained cells were analyzed using a C6 flow cytometer (Accuri, Ann Arbor, MI). Further histogram analyses were performed using FloJo software.

### RPE-EMT Induction

The hRPE monolayers were incubated in proteo-collagenolytic enzyme (AccuMAX, Sigma-Aldrich, St. Louis, MO) for 15 to 20 minutes. Gentle mechanical trituration was performed by pipetting approximately 15 times with a P1000 pipette. AccuMAX was neutralized by adding double the amount of RPE medium and AccuMAX was removed by aspiration after cells were centrifuged at 150×*g* for 5 minutes. Cell viability was assessed by Trypan blue dye exclusion. Cells were then resuspended in RPE medium and plated on Matrigel-coated plates at a density of 30,000 cells/cm^2^ and incubate at 37°C/5% CO_2_. For inducing TGF-β signaling associated RPE-EMT, the hRPE monolayers were cultured for approximately 3 months before cotreatment with equal concentration of 1 to 40 ng/mL of recombinant human TGF-β1 (Thermo Fisher Scientific, Waltham, MA; Catalog # PHG9204) and recombinant human TNF-α protein (R&D Systems. Minneapolis, MN; Catalog # 210-TA-020) in RPE medium for 24, 48, and 72 hours in 37°C/5% CO_2_.

### RNA Isolation and Quantitative RT-PCR

Quantitative PCRs were performed as described elsewhere^17^ and run in biological triplicates and expression levels normalized using the geometric mean of reference genes *GAPDH*, *ACTB*, *SRP72*, and *CREBBP*. Gene-specific primers sequences are presented in Supplementary Table S1.

### Immunostaining

The hRPE monolayers were fixed with 4% paraformaldehyde in PBS for 1 minute at room temperature, followed blocking with 5% goat serum and permeabilized with Triton X-100 in PBS for 30 minutes. Cells were labeled by incubation with the following primary antibodies—vimentin, TWIST1, ZO1, CLDN, RLBP1, TYR, RPE65, CDH1, and CDH2—for 2 hours at room temperature. Details about the primary antibodies and the dilutions that were used are given in Supplementary Table S2. Cells were stained with corresponding secondary antibody conjugated with Alexa Fluor 647 (Invitrogen) and nuclei were counterstained with Hoechst 33342 (Invitrogen). Images were acquired using an EVOS FL Auto Cell Imaging System (Life Technologies, Carlsbad, CA) and/or a Zeiss confocal microscope with 20X or 60X magnification. Images were processed using ImageJ software (National Institutes of Health, Bethesda, MD).

### RNA-Seq and Data Processing

First-strand cDNA synthesis was performed with 195 ng total RNA using anchored oligo-dT and SuperScript III First-Strand Synthesis SuperMix (Thermo Fisher Scientific). Second strand cDNA synthesis was performed using RNase H, DNA polymerase I, and Second Strand Buffer (Thermo Fisher Scientific). Double-stranded cDNA was purified using DNA Clean & Concentrator-5 (Zymo, Irvine, CA). Library preparation was performed using the Nextera XT DNA Library Preparation Kit (Illumina, San Diego, CA). Libraries were cleaned using Agencourt AMPure XP beads according to manufacturer's instructions (Beckman Coulter). Libraries were evaluated by the High Sensitivity DNA Kit on the 2100 Bioanalyzer. They were then multiplexed and sequenced on an Illumina HiSeq with 50 bp paired-end reads. Reads were aligned to NCBI build 37.2 using Tophat (v2.1.0).^22^ Cuffquant and Cuffnorm (Cufflinks v2.2.1) were used to quantify expression levels and calculate normalized fragments per kilobase of transcript per million mapped reads (FPKM) values.^23^

### RNA-Sequencing (RNA-Seq) Data Analysis

We performed Student *t* tests to find differentially expressed genes (DEGs). For enzymatic dissociation data, genes with log_2_ fold change of more than 1 and an adjusted *P* value of less than 0.3 were defined as DEGs. For TGF-β/TNF-α–induced EMT data, genes with log_2_ fold change of greater than 1 and an adjusted *P* value of less than 0.1 were defined as DEGs. For unsupervised hierarchical clustering, the Pearson correlation coefficient was used to construct the linkage matrix, and we used the Ward method for calculating distance between clusters.

### Biological Pathway and Upstream Regulator Analysis

Pathway analysis was performed using ingenuity pathway analysis (IPA) (Qiagen, Redwood City, CA). Transcripts that were identified to be differentially expressed during RPE-EMT compared with untreated monolayers with a greater than 2-fold change and a *P* value of less than 0.05 were input into IPA and KEGG for bioinformatics analysis using gene IDs. Differentially expressed transcripts were analyzed in IPA using core analysis followed by a comparison analysis between dissociation time course and TGF-β/TNF-α concentration. Datasets were assessed for prediction of canonical pathways and upstream regulators.^24^

### Statistical Analysis

All statistical analyzes were performed using Python data analytics program. The Fisher exact test, Student *t* test, one-way ANOVA, and Pearson's correlation coefficient were used to assess significance. Fold change, *P* values, and the false discovery rate were calculated in this analysis.

## Results

### Enzymatic Dissociation Induces EMT in hRPE Monolayers

**Figure 1.**
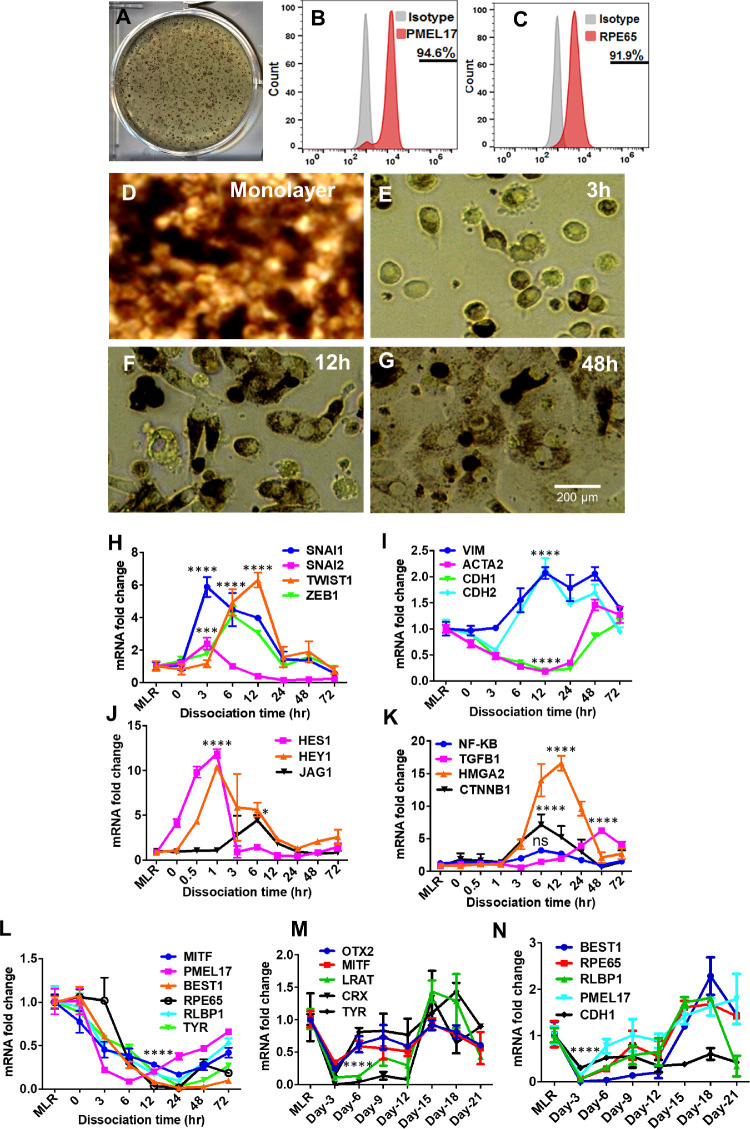
hRPE differentiation and enzymatic dissociation induced RPE-EMT. (**A**) Morphology of human pluripotent stem cells (hiPSC) with pigmented colonies after 45 days of differentiation. (**B**, **C**) Flow-cytometry of PMEL17 and RPE65 expression from 2-month-old RPE. (**D**) Bright field image of the 2-month-old hRPE monolayer with cobblestone morphology and (**E**, **G**) fibroblast morphology of hRPE cells after enzymatic dissociation induced EMT at 3 to 48 hours. (**H**–**K**) Differential expression of key EMT transcription factors, and its associated genes, differential expression of RPE-specific genes (**L**–**N**) were measured by qRT-PCR after enzymatic dissociation of monolayers (MLR) into single cells. Error bars represent the standard deviation of 3 biological replicates and statistically significant mean differences. Statistical comparisons between means were performed by a 2-tailed *t* test. A *P* value of 0.05 or less is considered as significant (symbol meaning: ns = *P* > 0.05, *P ≤ 0.05, **P ≤ 0.01, ***P ≤ 0.001, ****P ≤ 0.0001).

Human induced pluripotent stem cells were differentiated into mature RPE monolayers as previously described.^16^^,^^17^ During human pluripotent stem cells differentiation, typical pigmented colonies formed after 50 days in differentiation medium (Fig. 1A). Flow cytometry analysis showed more than 90% of the differentiating cells express RPE markers PMEL17and RPE65 (Figs. 1B and C). RPE colonies were then passaged twice and cultured for 2 to 3 months to obtain pure and mature RPE monolayers exhibiting typical RPE cobblestone morphology (Fig. 1D).

As one in vitro approach to model RPE-EMT, we used proteocollagenolytic enzymes (AccuMAX) to detach RPE monolayer cultures from their culture substrate and dissociated them into a single cell suspension and replated at a higher density (25,000 cells/cm^2^) on matrigel-coated culture plates. Enzymatically treated RPE cells lost their pigment and RPE-like morphologic characteristics and exhibited EMT-related phenotypic changes, including elongated fibroblast cell-like morphology (Figs. 1 E–G). Similar to the cells subjected to proteocollagenolytic enzyme treatment, we also detached hRPE cells using a cell scraper and replated them at an increased density (500,000/cm^2^). This method of cell harvesting, without enzymatic treatment, also showed differential expression of multiple EMT-associated factors and RPE-specific factors (Supplementary Figs. S6 and S7).

A quantitative RT-PCR analysis was used to assess whether changes in the expression of EMT-associated genes correlated with the observed changes in RPE morphology. We isolated mRNA directly from intact RPE monolayers as well as from acutely dissociated cells (0 hour) and from cells recultured for 3 to 72 hours after dissociation. *SNAI1* and *SNAI2*, 2 key EMT transcriptional regulators, showed an early increase in expression, increasing by 6- and 2-fold, respectively, over monolayer cultures at 3 hours after dissociation. Two other major EMT-related transcription factors—*ZEB1* and *TWIST1*—showed a 4- and 6-fold increased expression, respectively, but with slower kinetics of induction (Fig. 1H). The expression of EMT pathway genes that are known to be regulated by canonical EMT transcription factors was also assessed: *VIM* and *CDH2* were both upregulated by 12 hours after dissociation (2-fold), whereas other genes related to epithelial cell morphology such as *CDH1* and *ACTA2* were downregulated (5-fold) (Fig. 1I). An analysis of the transcripts of the genes further downstream of known canonical EMT signaling pathways also identified a number of highly significant increased expressions, including the NOTCH signaling-related genes *HES1*(12-fold), *HEY1* (10-fold), and *JAG1* (4-fold), as well as members of other signaling pathways, such as *TGFB1* (6-fold), *NFKB1* (3-fold), *HMGA2* (16-fold), and *CTNNB1* (7-fold) (Figs. 1J, K).

**Figure 2.**
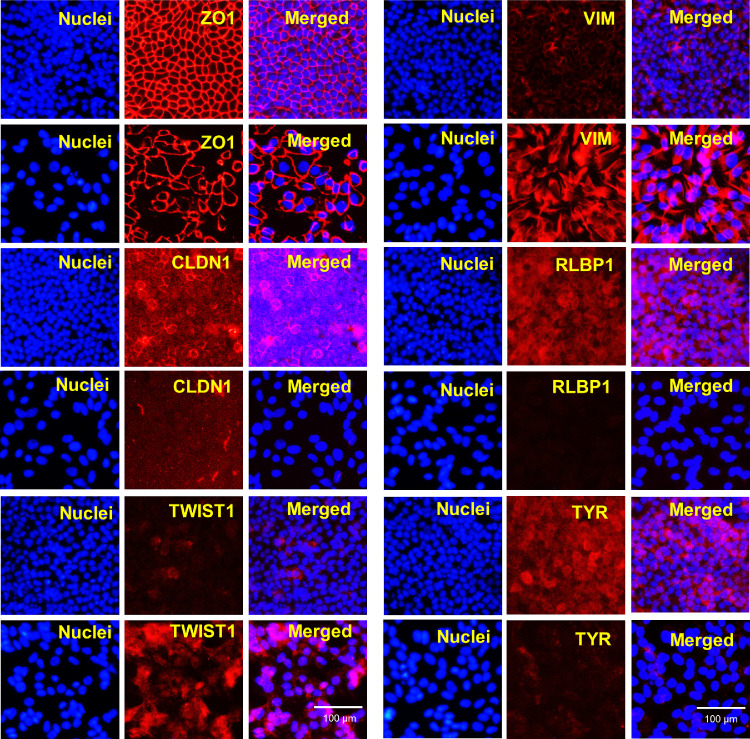
Immunofluorescence analysis of enzymatic dissociation induced RPE-EMT. hRPE monolayers were enzymatically dissociated and performed immunofluorescence. Dysregulated cytoskeletal changes (ZO-1, CLDN1, VIM), increased EMT transcription factor (TWIST1) and decreased RPE markers (RLBP1, TYR) (red) were shown by immunostained images. Hoechst 33342 was used to visualize the nuclei (blue). Scale bar, 100 µm.

To complement the analysis of EMT-related genes, we also looked at the expression of known RPE differentiation markers, because EMT is known to be associated with dedifferentiation, and observed marked and rapid downregulation of essentially all markers examined, including *MITF* (6-fold), *PMEL17* (12-fold), *BEST1* (76-fold), *RPE65* (61-fold), *RLBP1* (10-fold), and *TYR* (33-fold) (Fig. 1L). Further, we measured the expression patterns of these RPE transcripts at 2-day intervals for 21 days after enzymatic dissociation and observed that expression of early RPE transcripts (*MITF*, *PMEL17*, and *RLBP1*) were partially restored during this recovery period, but the expression of the late RPE markers, namely, *TYR*, *BEST1*, and *RPE65*, remained low during the entire 21-day period (Figs. 1M, N). Next, we validated these finding using another independent hiPS line (IMR 90.4) and observed similar differential expression of EMT-associated and RPE factors (Supplementary Fig. S4). We performed an immunofluorescence analysis of after dissociation induced RPE-EMT at 72 hours (Fig. 2), for multiple EMT factors that have been previously associated with malignancy. As expected, dissociation disrupted the tight junctions, showing a decreased and disorganized ZO1 and decreased expression of CLDN1. We observed an increase in the staining intensity of TWIST1 along with an increased and altered distribution of vimentin, changing from a localized perinuclear signal in mature hRPE monolayers to a diffuse and filamentous morphology. Further, we observed diminished staining intensity of the RPE factors RLBP1 and TYR. These protein expression changes correlate with the mRNA changes described elsewhere in this article. Taken together, our data indicate that the dissociation of hRPE monolayers leads to a process that shares many of the morphologic, gene expression, and protein expression changes that would be expected of RPE cells undergoing EMT.

### TGF-β/TNF-α Induces EMT in hRPE Monolayers

**Figure 3.**
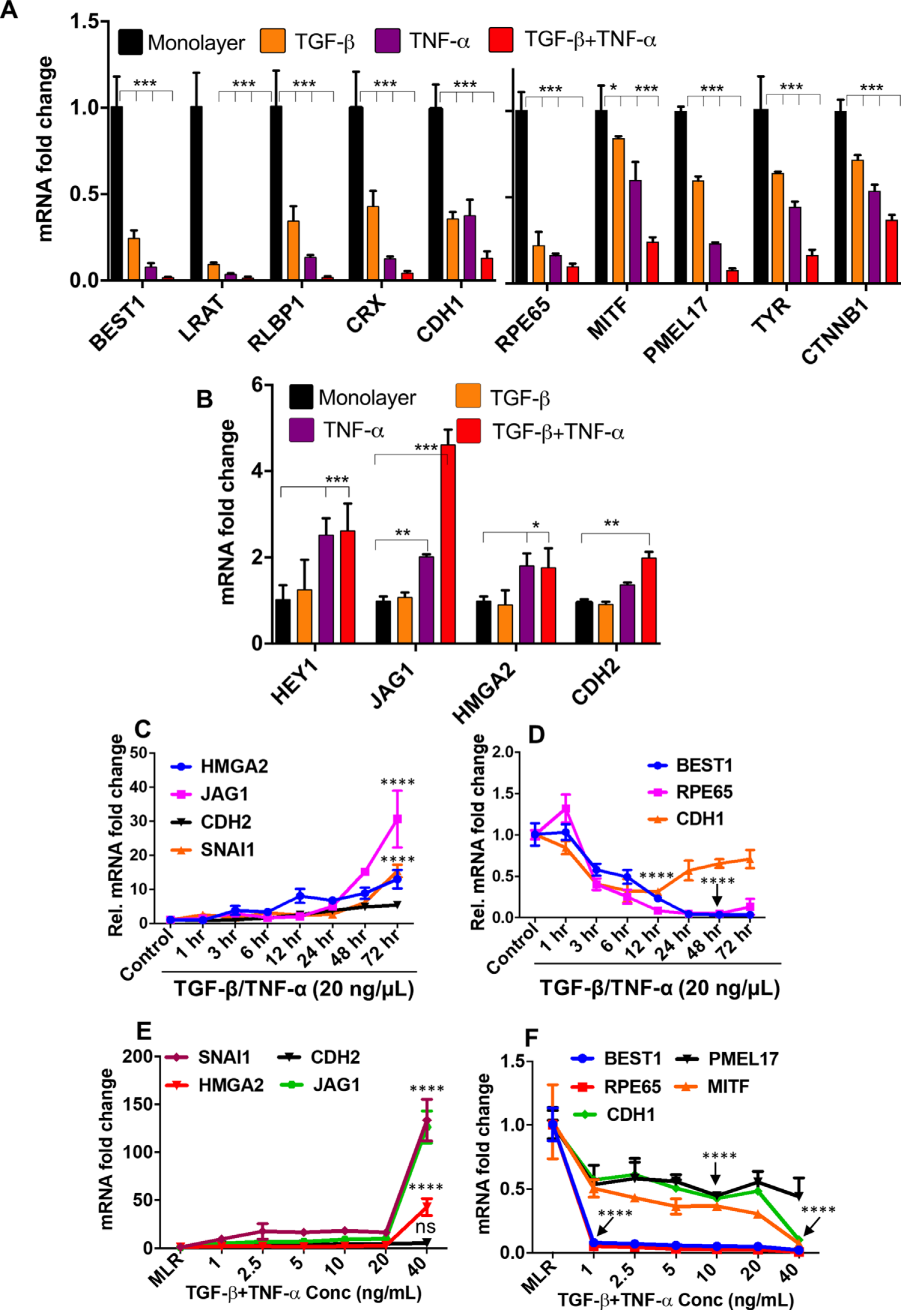
TGF-β/TNF-α cotreatment induces EMT in hRPE monolayers. hRPE monolayers treated with TGF-β only (40 ng/mL), TNF-α only (40 ng/mL), and TGF-β plus TNF-α (40 ng/mL) for 24 hours (EMT factors) and 72 hours (RPE factors) were analyzed by quantitative PCR for both (**A**) EMT-related and (**B**) RPE-specific genes. The time course (1–72 hours) expression of (**C**) EMT and (**D**) RPE transcripts after TGF-β/TNF-α treatment. Dose-dependent (1–40 ng/mL) TGF-β/TNF-α–induced expression of (**E**) EMT and (**F**) RPE transcripts. Error bars represent standard deviation of 3 biological replicates. Statistical comparisons between means were performed by a 2-tailed *t* test. A *P* value of 0.05 or less is considered as significant (symbol meaning: ns = *P* > 0.05, *P ≤ 0.05, **P ≤ 0.01, ***P ≤ 0.001, ****P ≤ 0.0001).

To broaden the generality of our studies and to assess the validity of the hRPE dissociation induced EMT model, we wanted to develop and analyze a second in vitro hRPE-EMT model. We first tested whether treatment with the known EMT inducer TGF-β^25^^,^^26^ could elicit gene expression changes consistent with EMT in the hRPE monolayer system. We treated hRPE monolayer cultures with TGF-β (40 ng/mL) or TNF-α (40 ng/mL) alone and also in combination with TGF-β and TNF-α^27^ for 1 to 72 hours, and then quantified the expression of mesenchymal and RPE transcripts. Although treatment with TGF-β or TNF-α alone led to some increased expression of EMT markers and a decreased expression of RPE markers and epithelial marker such as *CDH1*, cotreatment with TNF-α led to considerably greater effects (Figs. 3A, B). Next, we determined the dose and time dependence of the TGF-β/TNF-α–induced RPE-EMT response. RPE monolayers were treated with 20 ng/mL of TGF-β/TNF-α for 1 to 72 hours, and the expression of EMT and RPE-related transcripts were assessed by qRT-PCR.

There was a significant increase in the expression of EMT-related factors (Fig. 3C) and downregulation of RPE markers (Fig. 3D) starting after 24 hours and continuing through 72 hours after treatment. We next sought to optimize the concentration that would induce an EMT-like state. Because we observed the greatest EMT-related gene expression changes between 24 and 72 hours, we used this time frame to assess EMT and RPE marker gene expression after monolayer cultures were treated with a range of TGF-β/TNF-α concentrations between 1 and 40 ng/mL (Fig. 3E, F). The increased expression of mesenchymal factors and decreased expression of RPE factors seemed to almost be biphasic, with similar expression changes observed at TGF-β/TNF-α doses between 1 and 20 ng/mL, and then significiantly greater changes observed at the 40 ng/mL dose. Among the genes that showed significantly greater increases in expression at 40 ng/mL were *SNAI1* (133-fold), *HMGA2* (42-fold), *CDH2* (5-fold), and *JAG1* (126-fold); and those that showed greater decreases included *BEST1* (20-fold), *RPE65* (40-fold), *PMEL17* (1.8-old), *MITF* (3-fold), and *CDH1* (2-fold). We also validated these findings with a second iPSC line, IMR 90.4, confirming the ability of TGF-β/TNF-α to induce the differential expression of EMT-associated and RPE factors (Supplementary Fig. S5).

**Figure 4.**
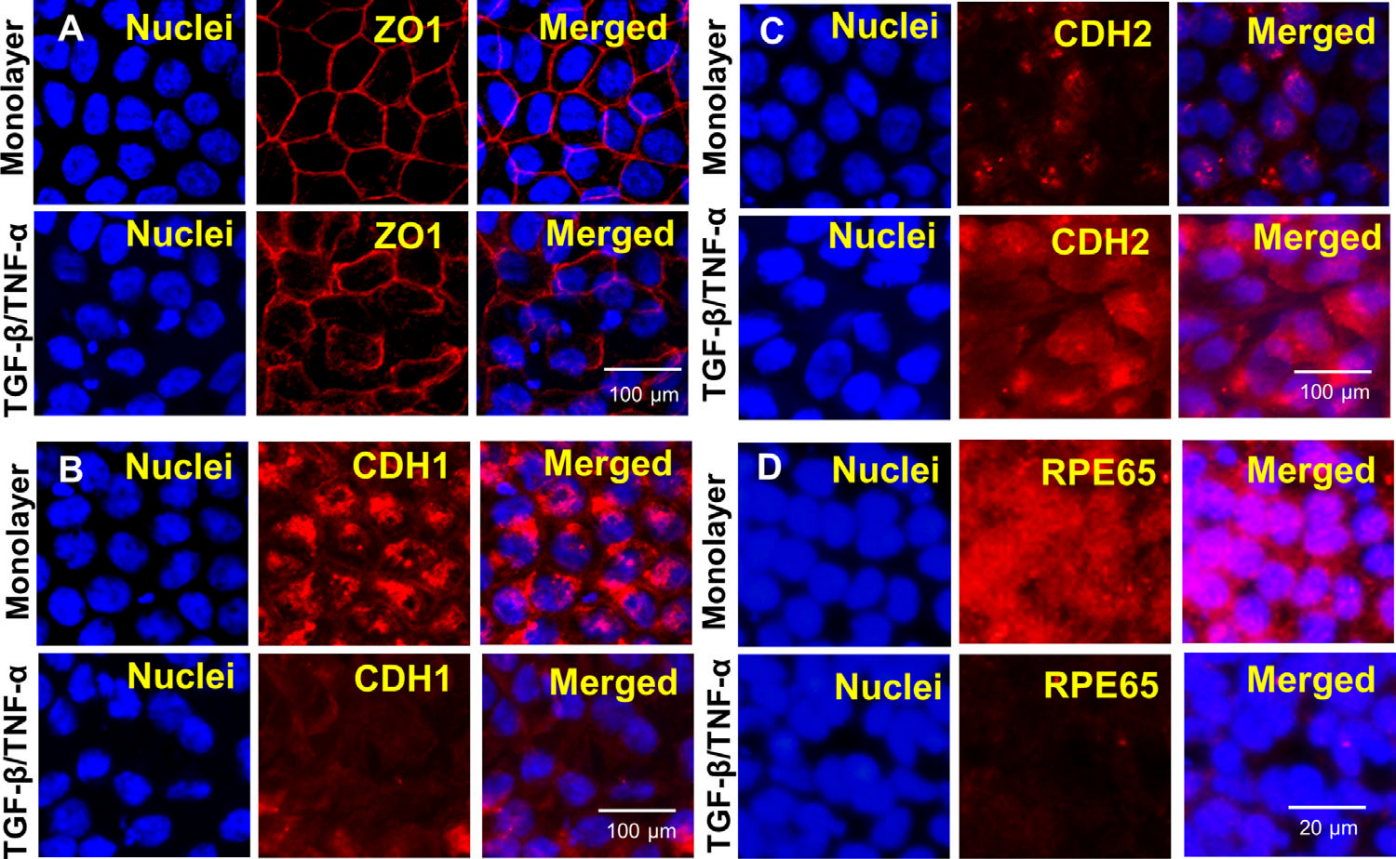
Immunofluorescence analysis of TGF-β/TNF-α–induced RPE-EMT. hRPE monolayers were cotreated with TGF-β and TNF-α (20 ng/mL) for 72 hours and immunostained for EMT and RPE factors. Disorganized tight junctions (ZO1) and dysregulated cadherin switch (increased expression of CDH2 and decreased expression of CDH1) were assessed by immunofluorescence (red). Hoechst 33342 was used to visualize the nuclei (blue). Scale bar, 20 µm. (H) Decreased expression of RPE65 (red) with cotreatment of TGF-β/TNF-α after 72 hours. Scale bar, 100 µm.

Prototypic epithelial monolayers are characterized by apical–basal polarity with lateral domains that have morphologically demarcated intercellular adhesive structures, such as tight junctions, with localized expression of *ZO1*.^28^ The deconstruction of tight junctions, with a loss of discrete ZO1 staining (Fig. 4A), is an early event during TGF-β–induced EMT.^29^ Another important aspect of TGF-β–induced EMT in cancer cells and in vitro epithelial cell EMT models is the cadherin switch, which is characterized by increased *CDH2* (N-cadherin) expression and decreased *CDH1* (E-cadherin) expression. The E/N cadherin switch occurs not only in cancer cells, but also in epithelial cells treated with TGF-β.^30^^,^^31^ Our immunofluorescence analysis confirmed similar loss of cell–cell junctional integrity and the cadherin switch following TGF-β/TNF-α–induced EMT. We observed disrupted tight junctions, increased intesity of *CDH2*, and decreased expression of *CDH1* from 72 hours after TGF-β/TNF-α treatment (Figs. 4B, C). Additionally, there was markedly decreased staining intensity for *RPE65* (Fig. 4D). These data indicate that TGF-β/TNF-α treatment effectively induces EMT in hRPE monolayers and provides a useful in vitro system for studying human RPE-EMT that complements the dissociation-induced EMT system as desctribed elsewhere in this article.

### Transcriptomic Changes Associated With hRPE-EMT Show Similarities With Malignancy-Associated EMT

To develop a comprehensive and publicly available resource of gene expression changes that occur during hRPE-EMT, we performed an RNA-seq analysis on cells subjected to EMT induction with enzymatic dissociation and TGF-β/TNF-α treatment. Dissociation-induced samples were harvested at 3, 12, and 48 hours after dissociation. For TGF-β/TNF-α samples, the hRPE monolayers were treated with doses of TGF-β/TNF-α between 1 and 40 ng/mL for 24 hours, at which time cells were harvested. We obtained an average of 13.4 million reads per sample from the dissociation samples, with an average mapping rate of 64.2% (human genome, NCBI build 37.2), and an average of 15 million reads per sample for the TGF-β/TNF-α treatments, with an average mapping rate of 53.11%. Principal component analysis shows the overall consistency of the replicates and the relative positions of the various experimental groups relative to the monolayer controls (Supplementary Figs. S3C, D).

**Figure 5.**
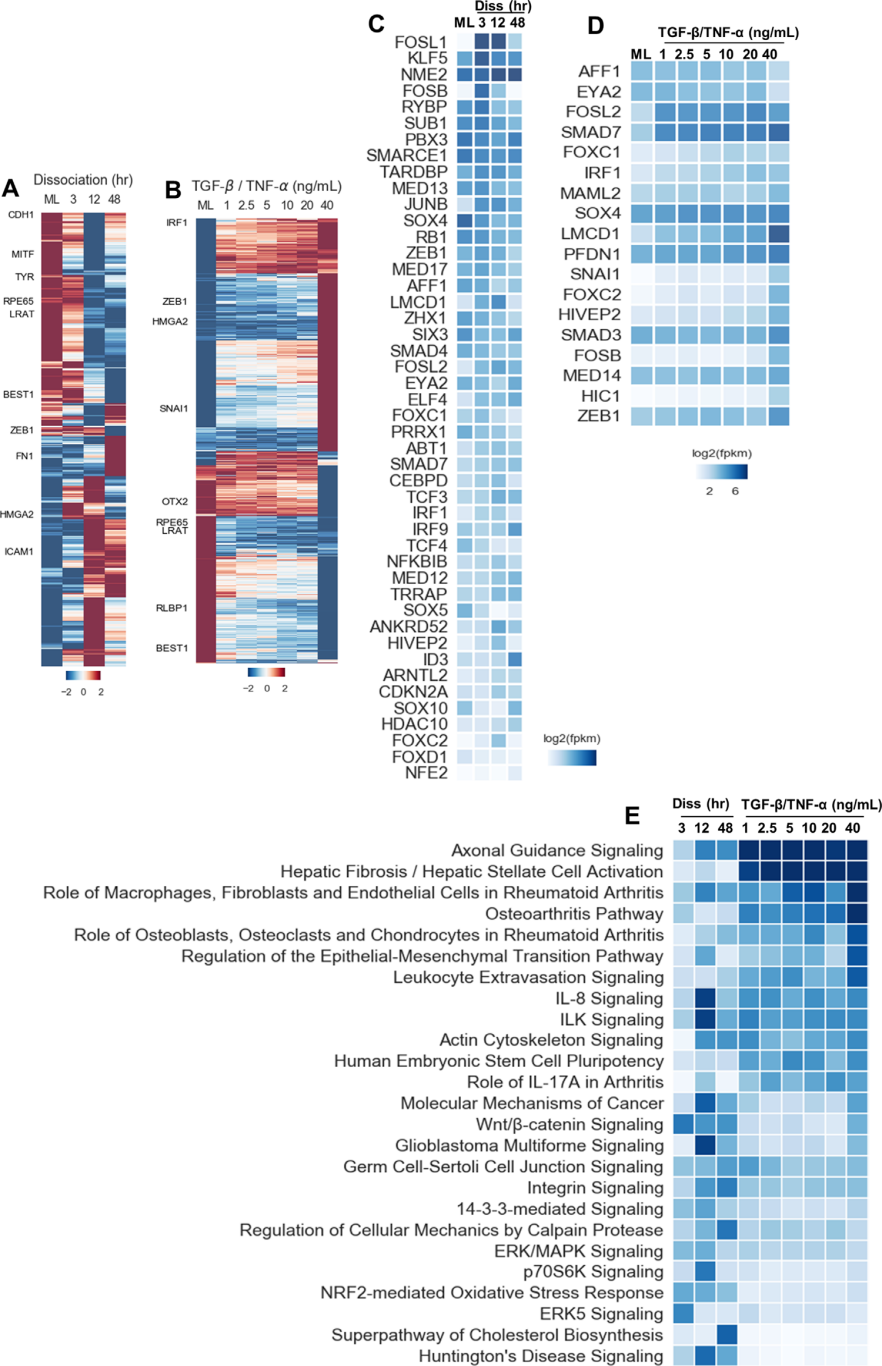
Temporal transcriptomic profiling of enzymatic dissociation and TGF-β/TNF-α–induced RPE-EMT. Hierarchical clustering of log_2_-transformed ratios and average abundances of DEG showing significant differences across time point after dissociation (**A**) and TGF-β/TNF-α (**B**) induced RPE-EMT. Previously reported transcription factors (TFs) and co-TFs that were significantly altered during enzymatic dissociation (**C**) and TGF-β/TNF-α (**D**) induced RPE-EMT. (**E**) Top canonical pathways were predicted based on the highly enriched genes that changed in abundance (activated or inhibited) during enzymatic dissociation and TGF-β/TNF-α induced EMT were plotted based on *P* values.

We identified 5397 (Dataset S01) and 1439 (Dataset S02) DEGs from enzymatic dissociation and TGF-β/TNF-α–induced RPE-EMT, respectively. Unsupervised hierarchical clustering of these DEGs in Figs. 5A and B showed distinct upregulated and downregulated patterns among different conditions in enzymatic dissociation and TGF-β/TNF-α–induced EMT. We found that 780 DEGs were common to both the dissociation and TGF-β/TNF-α EMT–induced samples (Supplementary Fig. S1A; Dataset 03). We plotted top 20 transcription factors that were upregulated (red) and downregulated (blue) during dissociation (Supplementary Fig. S2A) TGF-β/TNF-α–induced (Supplementary Fig. S2B) EMT. Further, we performed unsupervised hierarchical clustering of these DEGs (Supplementary Fig. S1B) and pathway enrichment analysis (Supplementary Fig. S1C). We next compared the transcriptional network correlation between our hRPE-EMT models and a previously studied mammary gland tumor associated model.^32^ We identified 46 genes (*P* = 1.5^–4^) and 18 genes (*P* = 1.02^–7^) from dissociation and TGF-β/TNF-α–induced EMT, respectively. As expected, we observered similarity between genes differentiated in the tumor and RPE-EMT models, particularly with genes known to be regulators of EMT and those involved in development, cell migration, focal adhesion, and integrin complex formation. Among these, we found that known cancer EMT-associated factors such as *SNAI1*, *ZEB1*, *FOSL*, *FOSB*, *JUNB*, *NFKB1*, *HIC1*, *SMAD3*, *FOXC2*, *LMCD1*, *ELF4*, and *IRF9* were upregulated, whereas the RPE factors *SOX4* and *SOX10* were downregulated significantly in dissociation and/or TGF-β/TNF-α–induced RPE-EMT (Figs. 5C, D).

### Dysregulated Axon Guidance Signaling Is Implicated in RPE-EMT

We next performed a gene ontology–based enrichment analyis of the differentially expressed RPE-EMT associated genes (Datasets S04, S05, and S06). One of the most significant, and potentially interesting, EMT-enriched canonical pathways was the axon guidance signaling pathway (dissociation: -log[*P* value] = 6.4; TGF-β/TNF-α: log[*P* value] = 14.8) (Fig. 5E). The second most enriched pathway arising from the IPA analysis was hepatic fibrosis/hepatic stellate cell activation, with enriched genes *COL8A2*, *MMP2*, *LAMA1*, *IGFBP5*, *PDGF*, *MYH8*, *TGFBR2*, and *SERPINE1*. Although, as the name implies, this pathway includes molecules related to response to liver damage, the molecules identified in these pathways also potentially modulate the tissue fibrosis that develops in the retina as part of the pathology associated with neovascular AMD.^33^ Other enriched pathways include EMT itself, the IL-8 pathway,^34^ the integrin-linked kinase (ILK) pathway,^35^ and the actin cytoskeleton signaling,^36^^,^^37^which are all known to contribute to cancer-related EMT.

**Figure 6.**
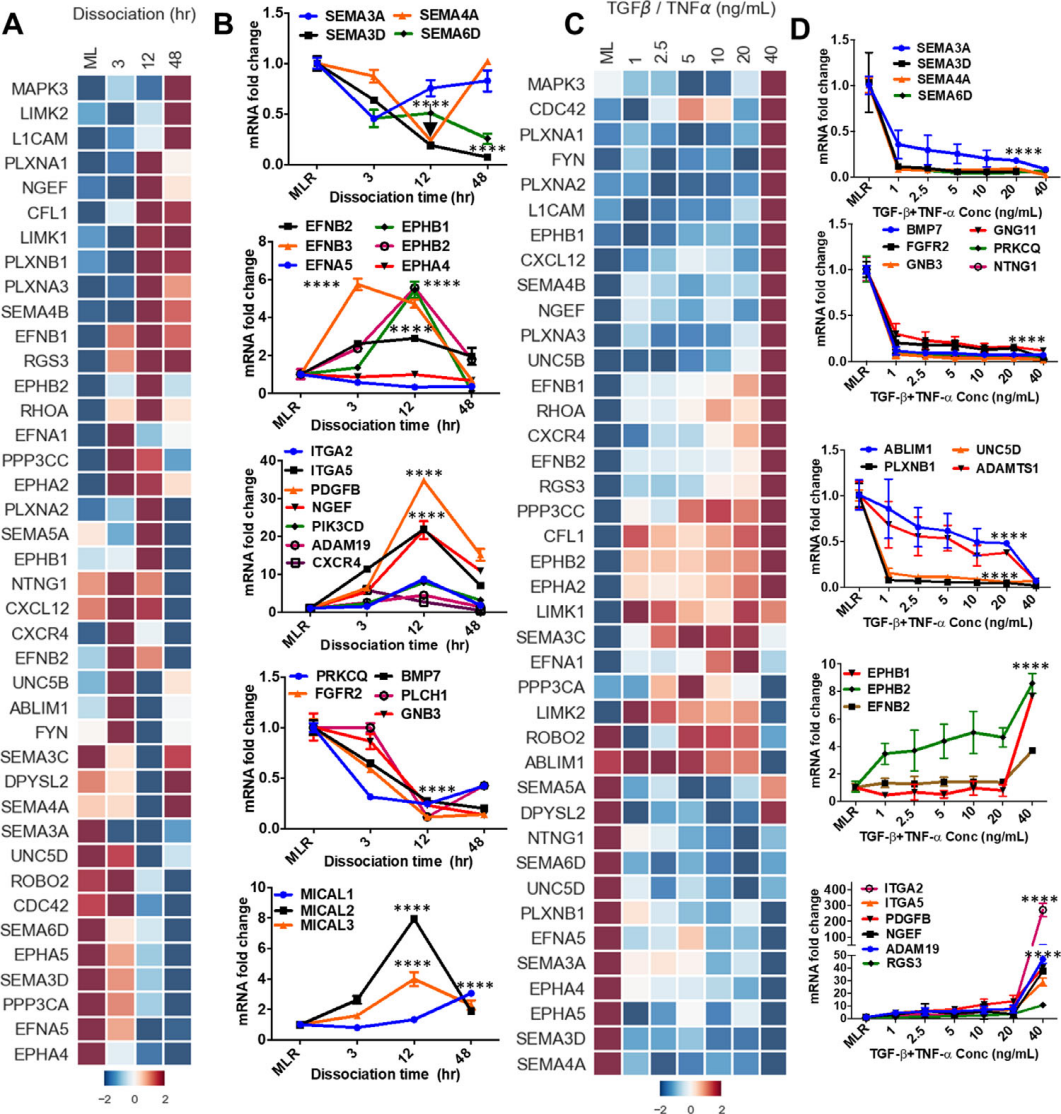
Dissociation and TGF-β/TNF-α–induced RPE-EMT induces altered axon guidance signaling. (**A**) Heatmap of IPA and KEGG identified top putative axon guidance molecules and (**B**) quantitative PCR validation of multiple dysregulated axon guidance genes from dissociation induced RPE-EMT. **(C**) Heatmap of IPA and KEGG identified top putative axon guidance molecules and (**D**) quantitative PCR validation of multiple dysregulated axon guidance genes from TGF-β/TNF-α–induced EMT. Error bars represent the standard deviation of 3 biological replicates and statistically significant mean differences. Statistical comparisons between means were performed by a 2-tailed *t* test. A *P* value of 0.05 or less was considered as significant (symbol meaning: ns = *P* > 0.05, *P ≤ 0.05, **P ≤ 0.01, ***P ≤ 0.001, ****P ≤ 0.0001).

To further define the changes that occur in axon guidance signaling during RPE-EMT, we specifically compared the transcriptome of highly enriched axon guidance genes from untreated RPE monolayers against EMT-induced RPE cells. Our RNA-seq analysis showed 40 transcripts of axon guidance-related factors that exhibit greater than 2-fold upregulation or downregulation (*P* < 0.05; ANOVA) (Figs. 6A, C). Next, we validated the dissociation-induced induced EMT changes of these axon guidance-related genes by qRT-PCR analysis. Genes encoding semaphorin family members (*SEMA3A*, *SEMA3D*, *SEMA4A*, and *SEMA6D*), which generally participate in the short range inhibition of axon growth cones, were significantly downregulated, whereas a subset of the ephrin axon guidance ligands (*EPHB1* and *EPHB2*), which interact with ephrins receptors (*EFNB1* and *EFNB2*), were upregulated during dissociation-induced RPE-EMT. However, the expression of *EFNA5* and *EPHA4* was not significantly changed. Additionally, several molecules interacting with CasL family members (*MICAL1*, *MICAL2*, and *MICAL3*), which are involved in axonal growth cone repulsion and actin cytoskeleton reorganization,^38^ were significantly upregulated during dissociation-induced RPE-EMT (Fig. 6B).

We also validated the differential expression of the axon guidance genes in the TGF-β/TNF-α RPE-EMT model (Fig. 6D). We found that *SEMA3A*, *SEMA3D*, *SEMA4A*, *SEMA6D*, *BMP7*, *FGFR2*, *GNB3*, *GNG11*, *PRKCQ*, *NTNG1*, *ABLIM1*, *UNC5D*, *PLXNB1*, and *ADAMTS1* were significantly downregulated by TGF-β/TNF-α treatment, and that *EPHB1*, *EPHB2*, *EFNB2*, *ITGA2*, *ITGA5*, *PDGFB*, *NGEF*, *ADAM19*, *RGS3*, and *MICAL2* were upregulated. Similar to our observations with some of the known EMT factors referred to elsewhere in this article, several of the axon guidance-related genes showed a nonlinear response to TGF-β/TNF-α concentration, showing a significant increase only at the 40 ng/mL dose. For example, *MAPK3*, *PLXNA1-3*, *EPHB1*, *CXCL12*, *SEMA4B*, *NGEF*, and *UNC5B* were upregulated only at 40 ng/mL. Conversely, *NTNG1*, *SEMA6D*, *UNC5D*, *PLXNB1*, *EFNA5*, *SEMA3A*, *EPHA4*, *EPHA5*, *SEMA3D*, and *SEMA4A* were downregulated only at 40 ng/mL. Further, we found that the axon guidance molecules *SEMA3A*, *SEMA3D*, *SEMA4A*, *SEMA6D*, *ITGA2*, *ITGA5*, *PDGFB*, *NGEF*, and *ADAM19* were altered in both EMT models; conversely, *EFNB2*, *EPHB1*, *EPHB2*, *PRKCQ*, *FGFR2*, and *BMP7* were only altered with dissociation, and*ABLIM1*, *PLXNB1*, *UNC5D*, and *ADAMTS1* were only altered with TGF-β/TNF-α treatment. To confirm the RNA-seq results, we performed quantitative PCR validation for select axon guidance genes using the same RNA samples that were used for the RNA-seq study, and noted tight correlation (Supplementary Fig. S3A, B). These observations suggest that multiple axon guidance molecules and pathways may be involved in RPE-EMT.

### Upstream Regulators of RPE-EMT

To investigate the cascade of transcriptional regulators that are upstream and presumably regulate the observed RPE-EMT DEGs, we analyzed the transcriptomic data using the “upstream regulator analysis” (URA) module in IPA. The URA algorithm uses overlap of p-value and an activation z-score to identify regulators that have been shown experimentally to ellicit gene expression patterns observed in a dataset.^24^ We filtered for regulators that were predicted to be activated or inhibited at least 1 time point from the dissociation EMT-induced samples (Dataset S07) and at least 1 dose from the TGF-β/TNF-α–induced EMT samples (Dataset S08) and from genes that overlapped from both EMT types (Supplementary Fig. S1D–J; Dataset S09). We clustered the resulting URs based on absolute value of the IPA activation score at any dissociation time/TGF-β/TNF-α dose to identify temporal and dose dependant regulated patterns. This analysis identified 3 broad categories of factors: (1) transcription factors that were previously reported to be involved in cancer-associated EMT progression, such as *NFKB1*,^39^ *STAT3*,^40^*RELA*,^41^*JUN*,^42^ and *HIF1A*;^43^ (2) transcription factors, most of which were downregulated, that are known to be involved in RPE differentiation, such as *OTX2*,^44^*BCL6*,^45^ and *ZFP36*,^46^ and (3) kinases that may modulate the RPE-EMT response, such as *AKT1*,^47^*MAPK1*,^48^*IKBKB*,^49^ and *MAP2K1* (*MEK1*).^50^ The URA analysis also predicted several growth factors andcytokines and enzymes as potential RPE-EMT regulators (Supplementary Figs. S1D–J, S2C–D). Together, the IPA generated URA suggests anRPE-EMT regulatory network and identifies putative master switches and nodes in its regulation.

### miRNAs as Regulators of RPE-EMT

Several studies have implicated noncoding miRNAs in retinal development and as important regulators of RPE-EMT.^51^^–^^53^ Although the methodology we used for RNA purification and library construction was designed for mRNA recovery and analysis, our analysis also identified several potential miRNAs that regulate RPE-EMT. Our data shows that miR22HG, miR155HG, miR77HG, and miR100HG (3 hours); miRLET7B and miR600HG (12 hours); and miR663HG (48 hours) were upregulated, whereas miR143HG (24 hours) was downregulated during dissociation induced RPE-EMT (Supplementary Fig. S2E, F). The URA analysis identified a number of miRNAs as candidate RPE-EMT modulators, including miR-1, miR-21, miR-26, miR-34, miR-122, miR-126, miR-135, miR-143, miR-145, miR-146, miR-210, miR-373 and, Let-7. The data, although incomplete, indicate that decreased expression of the miRNAs miR-373, miR-126, and let-7 was specifically associated with dissociation induced EMT; decreased expression of miR-515,miR-143, miR-135, and miR-1 was specifically associated with TGF-β/TNF-α–induced EMT; and decreased expression of miRNAs miR-34, miR-145, miR-146, and miR-126 was associated with both dissociation and TGF-β/TNF-α–induced RPE-EMT (Supplementary Fig. S2C, D). Of potential significance, mir-34 has been reported to be involved in the inhibition of proliferation and migration of RPE cells^54^ and miR-146 targets complement factor H, potentially modulating its expression in AMD.^55^ Taken together, these findings demonstrate that RPE-EMT is a complex process, regulated by multiple signaling networks and downstream effector pathways.

## Discussion

EMT is an important biological process that is involved in both normal tissue homeostasis and pathogenesis of a number of diseases.^32^^,^^56^ Here, we report transcriptional analysis of human RPE-associated EMT, studying EMT induced in hRPE monolayers using 2 independent but complimentary in vitro models: (1) enzymatic dissociation of monolayer cultures into single cells and (2) cotreatment of monolayer cultures with TGF-β and TNF-α. A gene ontology analysis of the observed RPE EMT-induced transcriptional changes demonstrated a strong and unexpected association of EMT with axonal guidance signaling and our data suggest the involvement of a ligand–receptor interaction between a number of key axonal guidance molecules. Consistent with this finding, although to our knowlege the overall axonal guidance pathway has not been implicated in REP-EMT previously, there have been a few previous studies that reported an association between individual axonal guidance molecules with cancer-related EMT and metastasis, including associations with semaphorin 3C, 3F, and 7A.^57^^–^^59^ A likely explanation is that molecules and pathways involved in the migration of neuronal cells and their neurites may also be used in the migration and process extension of RPE and other epithelial cells. TGF-β/TNF-α–induced RPE-EMT induces epigenomic changes and leads to fibrous epiretinal membranes that are similar to those observed with severe blinding disease conditions.^10^ Phenotypical EMT changes also occur during normal aging-associated conditions, which can affect tissue homeostasis and function.^60^ In addition, cellular senescence owing to aging can induce EMT in nonaggressive cancer cell lines.^61^ Excess RPE dedifferentiation can promote pathologic conditions associated with increased proliferation, which in turn can lead to a scarring, such as that seen in proliferative vitroretinopathy.^62^ Although our transcriptomic analysis implicates a broad range of signaling pathways that seem to contribute to RPE-EMT, each pathway might have distinct effects on the expression of specific RPE genes. For example, ILK signaling, which mediates several key events, including cell survival, proliferation, and differentiation, is enriched in dissociation-induced RPE-EMT. ILK signaling regulates the cross-talk between E-cadherin and integrin,^63^ and the increased expression of ILK results in the downregulation of E-cadherin through the activation of β-catenin and nuclear factor-κB.^64^ We observed that dissociation and TGF-β/TNF-α–induced RPE-EMT increased the abundance of E-cadherin, β-catenin, and nuclear factor-κB.

Recent studies reveal that RPE-EMT is also post-transcriptionally regulated by multiple noncoding miRNAs.^65^ For instance, URA of the RPE EMT datasets implicated several miRNAs already implicated in tumor invasion and metastasis, including miR-210 (ovarian cancer^66^), miR-122 (hepatocellular carcinoma^67^), miR146 (non-small cell lung cancer^68^), miR-124-3p (bladder cancer^69^), and miR-30c-5p (gastric cancer^70^). Together, our data establish a transcriptional hierarchy during enzymatic dissociation and TGF-β/TNF-α–induced RPE-EMT and identify number of master switches and nodes in its temporal and dose-dependent regulation.

In conclusion, we have delineated many of the regulatory interactions underlying both the enzymatic dissociation and TGF-β/TNF-α EMT models. The results identify several key RPE-EMT regulatory hubs, including axon guidance signaling and other key kinases, transcription factors, and miRNAs. We hope the extensive human RPE transcriptional datasets that we have generated, which are publicly available and can be explored interactively, will provide a useful resource to the vision research community for exploring the transcriptional changes and biological pathways involved in RPE-EMT. This information will hopefully aid in the development of therapeutics that focus on modulating the role of RPE-EMT in retinal disease. Additionally, developing the ability to inhibit EMT will have implications in the development of RPE transplantation-based therapies, because one of the challenges of safe and effective RPE transplantation is achieving cell purification and transfer without inducing EMT in either the donor or the host cells.^10^^,^^71^^,^^72^

## Supplementary Material

Supplement 1

Supplement 2

Supplement 3

Supplement 4

Supplement 5

Supplement 6

Supplement 7

Supplement 8

Supplement 9

Supplement 10

Supplement 11

Supplement 12
