# Supplementary material for: Transcriptome Landscape of Epithelial to Mesenchymal Transition of Human Stem Cell–Derived RPE
Source: Invest Ophthalmol Vis Sci. 2021 Apr 1;62(4):1. doi: 10.1167/iovs.62.4.1 (PMC8024778; doi:10.1167/iovs.62.4.1)
Supplement: Supplement 2 [file iovs-62-4-1_s002.pdf]

Figure S1

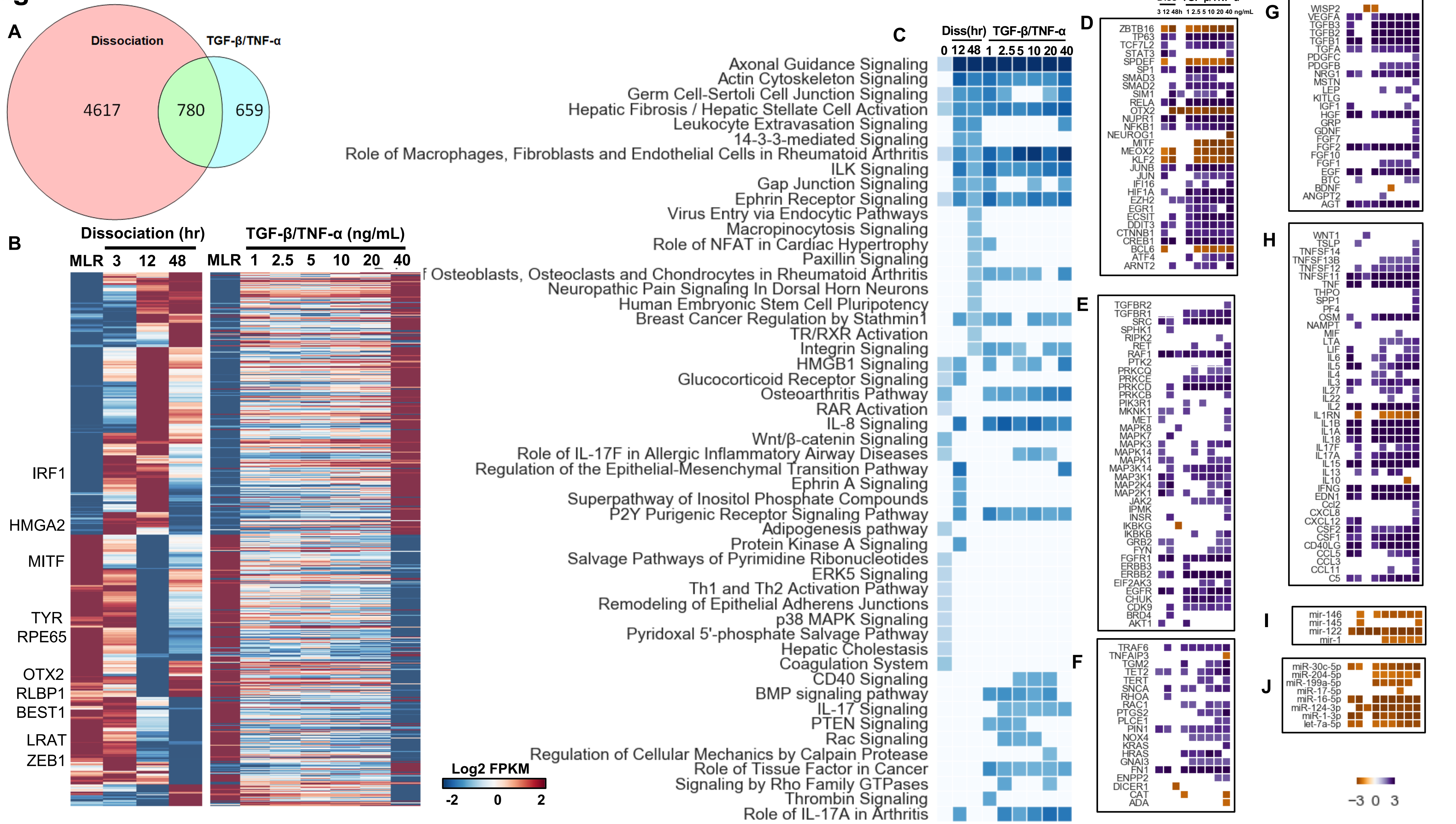

A
Dissociation
TGF-β/TNF-α
4617
780
659
B
Dissociation (hr)
TGF-β/TNF-α (ng/mL)
MLR 3 12 48
MLR 1 2.5 5 10 20 40
IRF1
HMGA2
MITF
TYR
RPE65
OTX2
RLBP1
BEST1
LRAT
ZEB1
Log2 FPKM
-2 0 2
C
Diss(hr) TGF-β/TNF-α
0 12 48 1 2.5 5 10 20 40
Axonal Guidance Signaling
Actin Cytoskeleton Signaling
Germ Cell-Sertoli Cell Junction Signaling
Hepatic Fibrosis / Hepatic Stellate Cell Activation
Leukocyte Extravasation Signaling
14-3-3-mediated Signaling
Role of Macrophages, Fibroblasts and Endothelial Cells in Rheumatoid Arthritis
ILK Signaling
Gap Junction Signaling
Ephrin Receptor Signaling
Virus Entry via Endocytic Pathways
Macropinocytosis Signaling
Role of NFAT in Cardiac Hypertrophy
Paxillin Signaling
f Osteoblasts, Osteoclasts and Chondrocytes in Rheumatoid Arthritis
Neuropathic Pain Signaling In Dorsal Horn Neurons
Human Embryonic Stem Cell Pluripotency
Breast Cancer Regulation by Stathmin1
TR/RXR Activation
Integrin Signaling
HMGB1 Signaling
Glucocorticoid Receptor Signaling
Osteoarthritis Pathway
RAR Activation
IL-8 Signaling
Wnt/β-catenin Signaling
Role of IL-17F in Allergic Inflammatory Airway Diseases
Regulation of the Epithelial-Mesenchymal Transition Pathway
Ephrin A Signaling
Superpathway of Inositol Phosphate Compounds
P2Y Purigenic Receptor Signaling Pathway
Adipogenesis pathway
Protein Kinase A Signaling
Salvage Pathways of Pyrimidine Ribonucleotides
ERK5 Signaling
Th1 and Th2 Activation Pathway
Remodeling of Epithelial Adherens Junctions
p38 MAPK Signaling
Pyridoxal 5'-phosphate Salvage Pathway
Hepatic Cholestasis
Coagulation System
CD40 Signaling
BMP signaling pathway
IL-17 Signaling
PTEN Signaling
Rac Signaling
Regulation of Cellular Mechanics by Calpain Protease
Role of Tissue Factor in Cancer
Signaling by Rho Family GTPases
Thrombin Signaling
Role of IL-17A in Arthritis
D
Diss TGF-β/TNF-α
3 12 48h 1 2.5 5 10 20 40 ng/mL
ZBTB16
TP63
TCF7L2
STAT3
SPDEF
SP1
SMAD3
SMAD2
SIM1
RELA
OTX2
NUPR1
NFKB1
NEUROG1
MITF
MEOX2
KLF2
JUNB
JUN
IFI16
HIF1A
EZH2
EGR1
ECSIT
DDIT3
CTNNB1
CREB1
BCL6
ATF4
ARNT2
E
TGFBR2
TGFBR1
SRC
SPHK1
RIPK2
RET
RAF1
PTK2
PRKCQ
PRKCE
PRKCD
PRKCB
PIK3R1
MKNK1
MET
MAPK8
MAPK7
MAPK3
MAPK14
MAPK1
MAP3K14
MAP3K1
MAP2K4
MAP2K1
JAK2
IPMK
INSR
IKBKG
IKBKB
GRB2
FYN
FGFR1
ERBB3
ERBB2
EIF2AK3
EGFR
CHUK
CDK9
BRD4
AKT1
F
TRAF6
TNFAIP3
TGM2
TET2
TERT
SNCA
RHOA
RAC1
PTGS2
PLCE1
PIN1
NOX4
KRAS
HRAS
GNAI3
FN1
ENPP2
DICER1
CAT
ADA
G
Diss TGF-β/TNF-α
3 12 48h 1 2.5 5 10 20 40 ng/mL
WISP2
VEGFA
TGFB3
TGFB2
TGFB1
TGFA
PDGFC
PDGFB
NRG1
MSTN
LEP
KITLG
IGF1
HGF
GRP
GDNF
FGF7
FGF2
FGF10
FGF1
EGF
BTC
BDNF
ANGPT2
AGT
H
WNT1
TSLP
TNFSF14
TNFSF13B
TNFSF12
TNFSF11
TNF
THPO
SPP1
PF4
OSM
NAMPT
MIF
LTA
LIF
IL6
IL5
IL4
IL3
IL27
IL22
IL2
IL1RN
IL1B
IL1A
IL18
IL17F
IL17A
IL15
IL13
IL10
IFNG
EDN1
Ccl2
CXCL8
CXCL12
CSF2
CSF1
CD40LG
CCL5
CCL3
CCL11
C5
I
mir-146
mir-145
mir-122
mir-1
J
miR-30c-5p
miR-204-5p
miR-199a-5p
miR-17-5p
miR-16-5p
miR-124-3p
miR-1-3p
let-7a-5p
-3 0 3

Figure S2

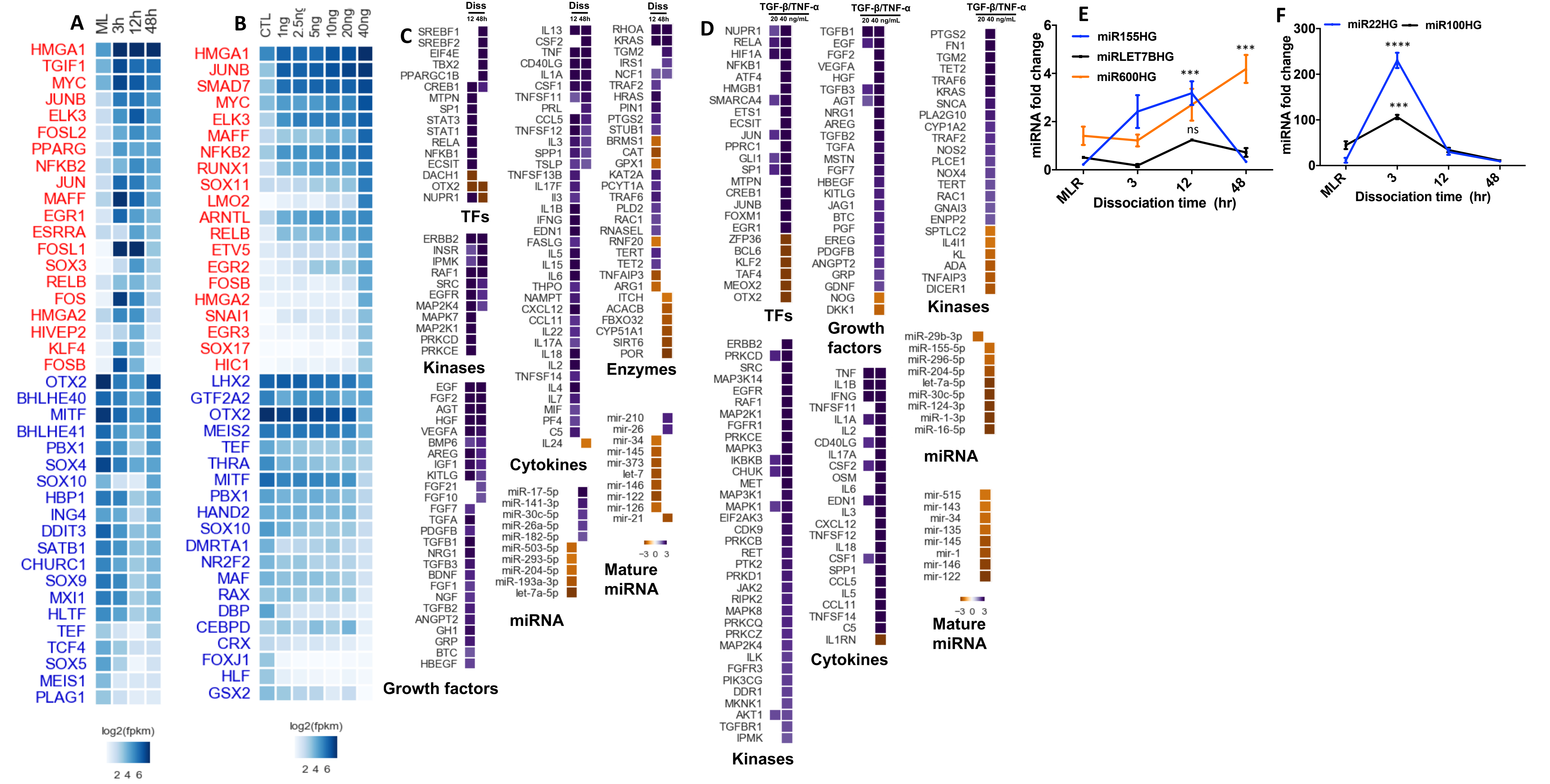

A
ML 3h 12h 48h
HMGA1 TGIF1 MYC JUNB ELK3 FOSL2 PPARG NFKB2 JUN MAFF EGR1 ESRRA FOSL1 SOX3 RELB FOS HMGA2 HIVEP2 KLF4 FOSB
OTX2 BHLHE40 MITF BHLHE41 PBX1 SOX4 SOX10 HBP1 ING4 DDIT3 SATB1 CHURC1 SOX9 MXI1 HLTF TEF TCF4 SOX5 MEIS1 PLAG1
log2(fpkm)
2 4 6
B
CTL 1ng 2.5ng 5ng 10ng 20ng 40ng
HMGA1 JUNB SMAD7 MYC ELK3 MAFF NFKB2 RUNX1 SOX11 LMO2 ARNTL RELB ETV5 EGR2 FOSB HMGA2 SNAI1 EGR3 SOX17 HIC1
LHX2 GTF2A2 OTX2 MEIS2 TEF THRA MITF PBX1 HAND2 SOX10 DMRTA1 NR2F2 MAF RAX DBP CEBPD CRX FOXJ1 HLF GSX2
log2(fpkm)
2 4 6
C
Diss
12 48h
SREBF1 SREBF2 EIF4E TBX2 PPARGC1B CREB1 MTPN SP1 STAT3 STAT1 RELA NFKB1 ECSIT DACH1 OTX2 NUPR1
TFs
ERBB2 INSR IPMK RAF1 SRC EGFR MAP2K4 MAPK7 MAP2K1 PRKCD PRKCE
Kinases
EGF FGF2 AGT HGF VEGFA BMP6 AREG IGF1 KITLG FGF21 FGF10 FGF7 TGFA PDGFB TGFB1 NRG1 TGFB3 BDNF FGF1 NGF TGFB2 ANGPT2 GH1 GRP BTC HBEGF
Growth factors
IL13 CSF2 TNF CD40LG IL1A CSF1 TNFSF11 PRL CCL5 TNFSF12 IL3 SPP1 TSLP TNFSF13B IL17F Il3 IL1B IFNG EDN1 FASLG IL5 IL15 IL6 THPO NAMPT CXCL12 CCL11 IL22 IL17A IL18 IL2 TNFSF14 IL4 IL7 MIF PF4 C5 IL24
Cytokines
miR-17-5p miR-141-3p miR-30c-5p miR-26a-5p miR-182-5p miR-503-5p miR-293-5p miR-204-5p miR-193a-3p let-7a-5p
miRNA
RHOA KRAS TGM2 IRS1 NCF1 TRAF2 HRAS PIN1 PTGS2 STUB1 BRMS1 CAT GPX1 KAT2A PCYT1A TRAF6 PLD2 RAC1 RNASEL RNF20 TERT TET2 TNFAIP3 ARG1 ITCH ACACB FBXO32 CYP51A1 SIRT6 POR
Enzymes
mir-210 mir-26 mir-34 mir-145 mir-373 let-7 mir-146 mir-122 mir-126 mir-21
−3 0 3
Mature miRNA
D
TGF-β/TNF-α
20 40 ng/mL
NUPR1 RELA HIF1A NFKB1 ATF4 HMGB1 SMARCA4 ETS1 ECSIT JUN PPRC1 GLI1 SP1 MTPN CREB1 JUNB FOXM1 EGR1 ZFP36 BCL6 KLF2 TAF4 MEOX2 OTX2
TFs
ERBB2 PRKCD SRC MAP3K14 EGFR RAF1 MAP2K1 FGFR1 PRKCE MAPK3 IKBKB CHUK MET MAP3K1 MAPK1 EIF2AK3 CDK9 PRKCB RET PTK2 PRKD1 JAK2 RIPK2 MAPK8 PRKCQ PRKCZ MAP2K4 ILK FGFR3 PIK3CG DDR1 MKNK1 AKT1 TGFBR1 IPMK
Kinases
TGFB1 EGF FGF2 VEGFA HGF TGFB3 AGT NRG1 AREG TGFB2 TGFA MSTN FGF7 HBEGF KITLG JAG1 BTC PGF EREG PDGFB ANGPT2 GRP GDNF NOG DKK1
Growth factors
TNF IL1B IFNG TNFSF11 IL1A IL2 CD40LG IL17A CSF2 OSM IL6 EDN1 IL3 CXCL12 TNFSF12 IL18 CSF1 SPP1 CCL5 IL5 CCL11 TNFSF14 C5 IL1RN
Cytokines
PTGS2 FN1 TGM2 TET2 TRAF6 KRAS SNCA PLA2G10 CYP1A2 TRAF2 NOS2 PLCE1 NOX4 TERT RAC1 GNAI3 ENPP2 SPTLC2 IL4I1 KL ADA TNFAIP3 DICER1
Kinases
miR-29b-3p miR-155-5p miR-296-5p miR-204-5p let-7a-5p miR-30c-5p miR-124-3p miR-1-3p miR-16-5p
miRNA
mir-515 mir-143 mir-34 mir-135 mir-145 mir-1 mir-146 mir-122
−3 0 3
Mature miRNA
E
miR155HG
miRLET7BHG
miR600HG
miRNA fold change
0 2 4 6
***
***
ns
MLR 3 12 48
Dissociation time (hr)
F
miR22HG
miR100HG
miRNA fold change
0 100 200 300
****
***
MLR 3 12 48
Dissociation time (hr)

Figure S3

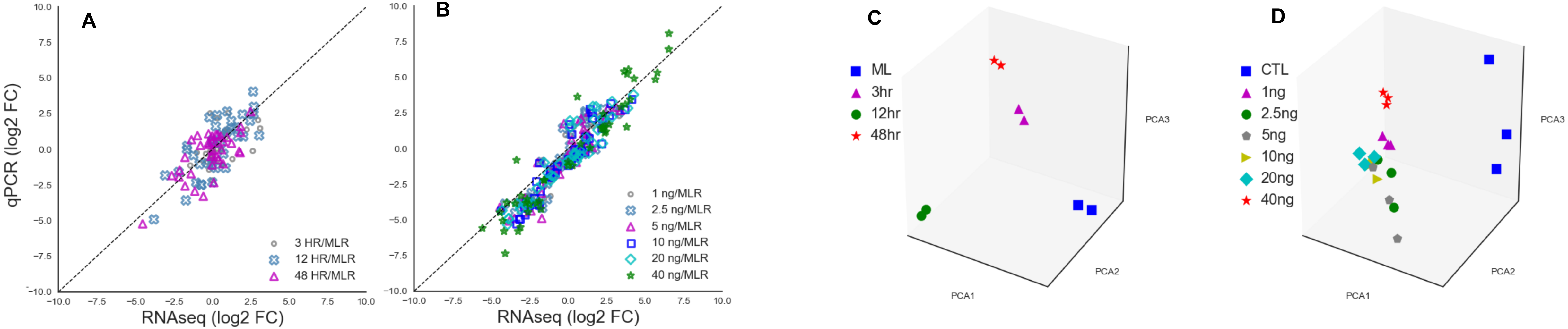
A
B
C
D
qPCR (log2 FC)
RNAseq (log2 FC)
3 HR/MLR
12 HR/MLR
48 HR/MLR
1 ng/MLR
2.5 ng/MLR
5 ng/MLR
10 ng/MLR
20 ng/MLR
40 ng/MLR
ML
3hr
12hr
48hr
CTL
1ng
2.5ng
5ng
10ng
20ng
40ng
PCA1
PCA2
PCA3

D

Figure S4

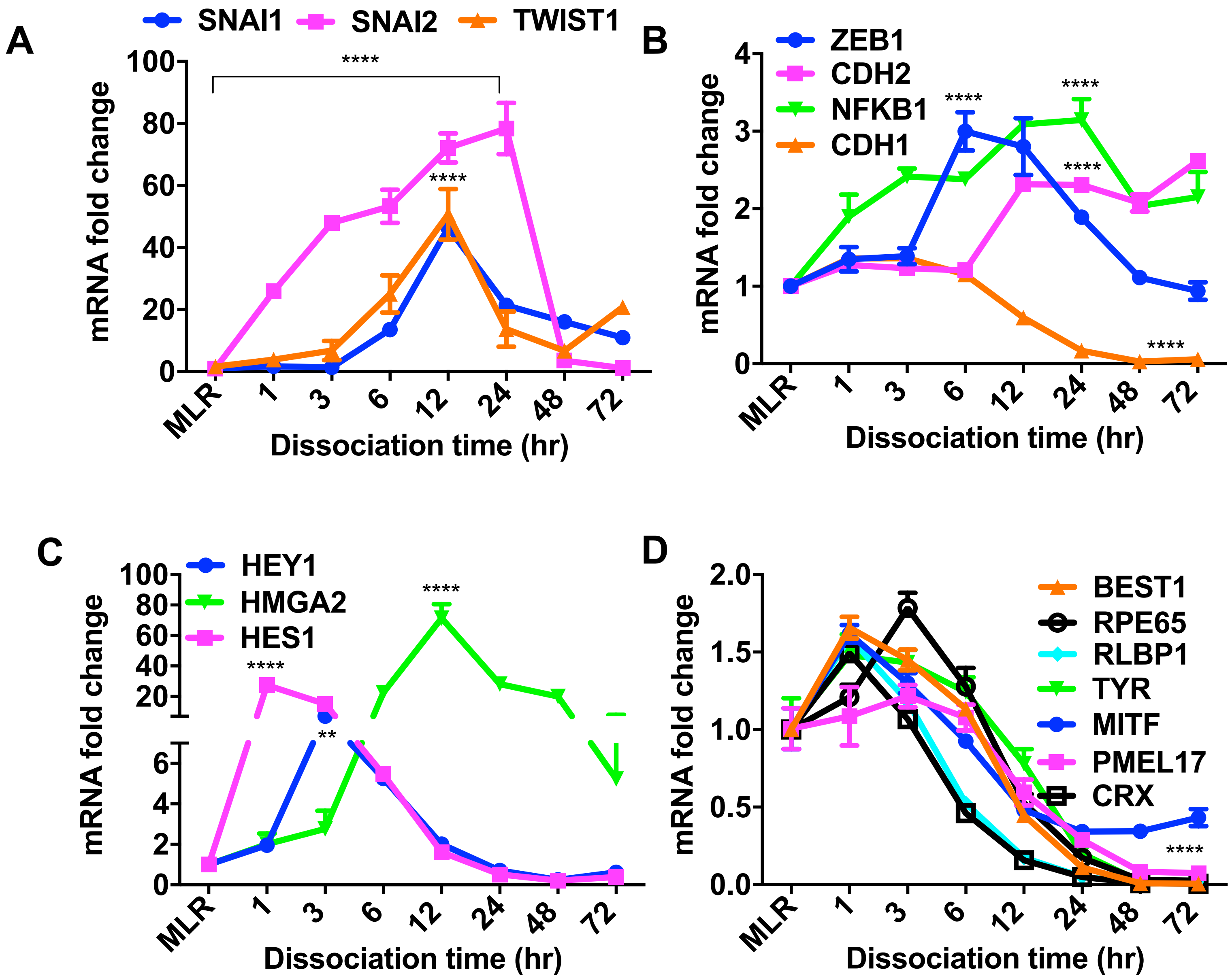
A
SNAI1
SNAI2
TWIST1
mRNA fold change
100
80
60
40
20
0
****
****
MLR
1
3
6
12
24
48
72
Dissociation time (hr)
B
ZEB1
CDH2
NFKB1
CDH1
mRNA fold change
4
3
2
1
0
****
****
****
****
MLR
1
3
6
12
24
48
72
Dissociation time (hr)
C
HEY1
HMGA2
HES1
mRNA fold change
100
80
60
40
20
6
4
2
0
****
****
**
MLR
1
3
6
12
24
48
72
Dissociation time (hr)
D
BEST1
RPE65
RLBP1
TYR
MITF
PMEL17
CRX
mRNA fold change
2.0
1.5
1.0
0.5
0.0
****
MLR
1
3
6
12
24
48
72
Dissociation time (hr)

Figure S5

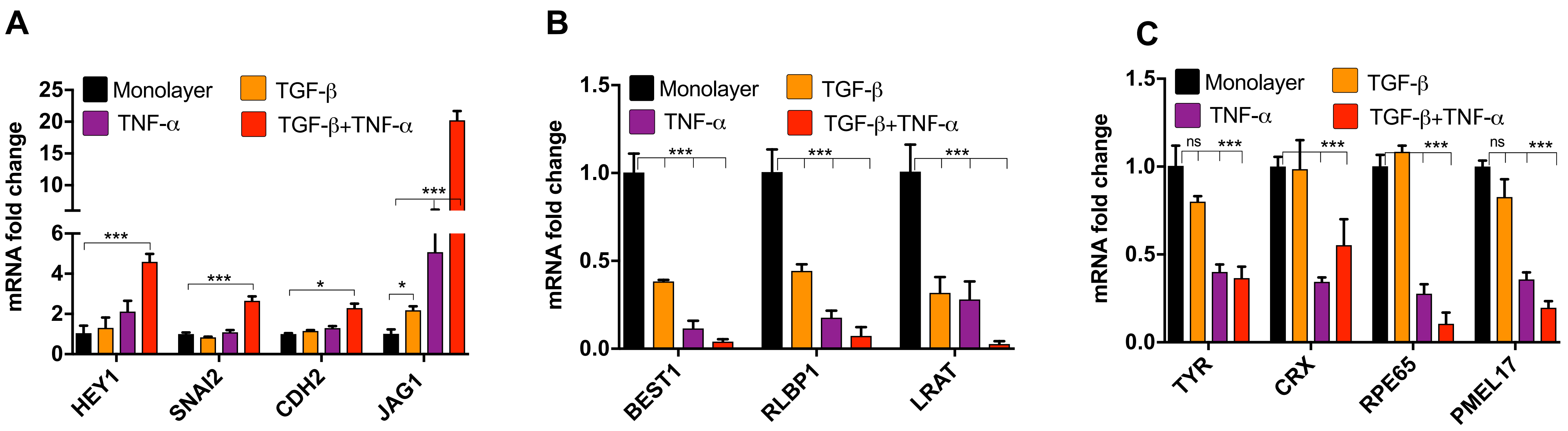
A
B
C
Monolayer
TGF-β
TNF-α
TGF-β+TNF-α
mRNA fold change
HEY1
SNAI2
CDH2
JAG1
BEST1
RLBP1
LRAT
TYR
CRX
RPE65
PMEL17
ns
***
*

Figure S6

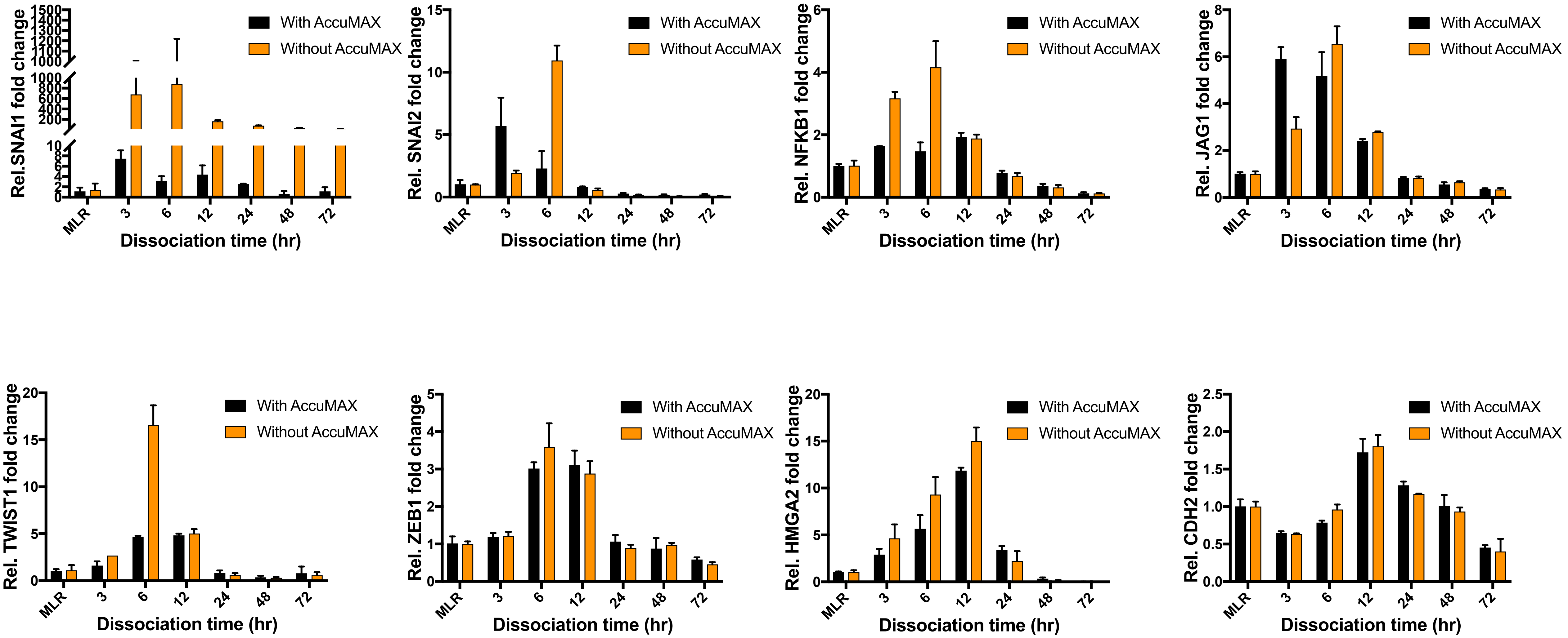

With AccuMAX
Without AccuMAX
Rel.SNAI1 fold change
Rel. SNAI2 fold change
Rel. NFKB1 fold change
Rel. JAG1 fold change
Rel. TWIST1 fold change
Rel. ZEB1 fold change
Rel. HMGA2 fold change
Rel. CDH2 fold change
MLR
3
6
12
24
48
72
Dissociation time (hr)

Figure S7

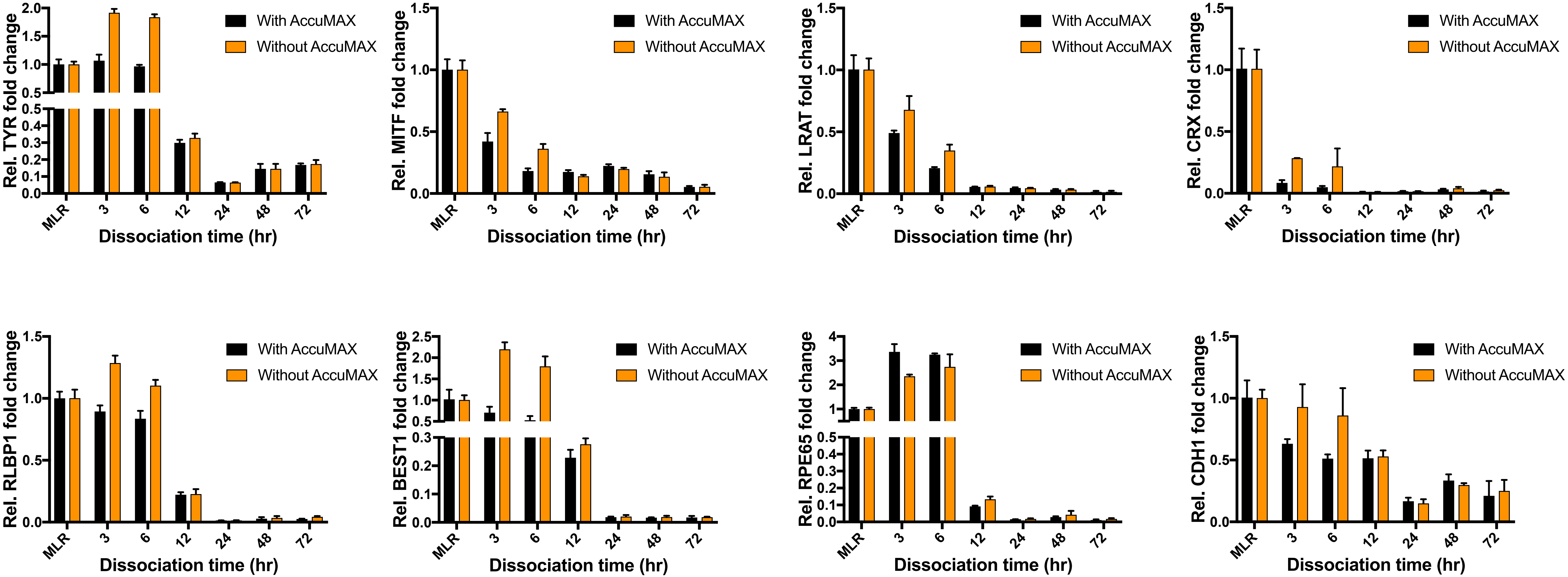

Rel. TYR fold change
Rel. MITF fold change
Rel. LRAT fold change
Rel. CRX fold change
Rel. RLBP1 fold change
Rel. BEST1 fold change
Rel. RPE65 fold change
Rel. CDH1 fold change
With AccuMAX
Without AccuMAX
MLR
3
6
12
24
48
72
Dissociation time (hr)

Figure S1. Transcriptomic profiling shows commonality between enzymatic dissociation and TGF-β/TNF-α induced RPE EMT.

(A) Venn diagram represents the genes that shares the common number of genes between dissociation (3-48 hr) and TGF-β/TNF-α (1-40 ng/mL) induced EMT-RPE. (B) Hierarchical clustering of log_2-transformed ratios and average abundances of differentially expressed genes showing significant differences across time point after enzymatic dissociation and TGF-β/TNF-α induced RPE-EMT. (C) Top canonical pathways were predicted based on the highly enriched genes that changed in abundance (activated or inhibited) during enzymatic dissociation and TGF-β/TNF-α induced EMT were plotted based on p-value. (D-J) Heatmaps of IPA generated upstream regulator analysis from common genes that altered from both RPE-EMT. IPA uses activation Z-score as a statistical measure of the match between expected relationship direction and observed changes in gene expression regulator including (D) transcription regulators (E) kinases (F) enzymes(G) growth factors (H) cytokines (I-J) miRNAs.

Figure S2. Top transcription factors and upstream regulators of enzymatic dissociation and TGF-β/TNF-α induced RPE-EMT.

Heat maps of top 20 transcription factors that up-regulated (EMT) and down-regulated (RPE) during enzymatic dissociation induced (A) and TGF-β/TNF-α (B) induced RPE-EMT. (C) Heatmaps of IPA generated upstream regulators identified from dissociation (C) and TGF-β/TNF-α (D) induced RPE-EMT. (E-F) Expression ratios for miRNA were plotted against log_2 fold change value from the RNA-seq analysis of dissociation induced RPE-EMT.

Figure S3. Correlation of RPE EMT gene expression ratios between RNA-seq and qRT PCR.

The expression ratios were plotted against log_2 fold change value from RNA-seq (x-axis) and qRT PCR was performed using the amplified cDNA from each RNAseq sample (y-axis). Statistically significant Pearson correlation is shown between the gene expression levels analyzed by qRT PCR and RNA-seq (A) Dissociation/Monolayer (39 genes), (B) TGF-B/TNF-A/Monolayer (54 genes). Principal Components Analysis (PCA) of gene expression data from dissociation (C) and TGF-

β/TNF-α treatment (D) were significantly correlated. Only genes with statistically significant changes at FDR of 5% (q<0.05) were included in analysis.

Figure S4. Validation of dissociation induced RPE-EMT in hiPS (IMR90.4) RPE cells. Differential expression of key EMT associated genes (A-C) and RPE specific genes (D) were measured by qRT PCR after enzymatic dissociation of monolayers (MLR) into single cells.

Figure S5. Validation of TGF-β/TNF-α induced RPE-EMT in hiPS (IMR90.4) RPE cells. Differential expression of key EMT associated genes (A-B) and RPE specific genes (C) were measured by qRT PCR after enzymatic dissociation of monolayers (MLR) into single cells.

Figure S6. Enzyme-free dissociation induced RPE-EMT. Differential expression of EMT-associated factor due to the physical (using cell scraper) dissociation.

Figure S7. **Enzyme-free dissociation induced RPE-EMT.** Differential expression of RPE-associated factor due to the physical (using cell scraper) dissociation.

Dataset S01. List of genes that enriched during dissociation induced RPE-EMT

Dataset S02. List of genes that enriched during TGF-β/TNF-α RPE EMT induced RPE-EMT

Dataset S03. List of genes that altered from both dissociation and TGF-β/TNF-α induced RPE-EMT

Dataset S04. List of canonical pathways that enriched during dissociation induced RPE EMT

Dataset S05. List of canonical pathways that enriched during TGF-β/TNF-α RPE EMT

Dataset S06. List of canonical pathways that enriched in common from dissociation and TGF-β/TNF-α induced RPE-EMT

Dataset S07. Upstream regulator analysis of enzymatic dissociation induced RPE EMT

Dataset S08. Upstream regulator analysis of TGF-β/TNF-α induced RPE EMT

Dataset S09. Upstream regulators that enriched in common from dissociation and TGF-β/TNF-α induced RPE-EMT.
