# Supplementary material for: Transcriptome Landscape of Epithelial to Mesenchymal Transition of Human Stem Cell–Derived RPE
Source: Invest Ophthalmol Vis Sci. 2021 Apr 1;62(4):1. doi: 10.1167/iovs.62.4.1 (PMC8024778; doi:10.1167/iovs.62.4.1)
Supplement: Supplement 3 [file iovs-62-4-1_s003.pdf]

Supplementary Table 1. Quantitative real time PCR sequence list

Quantitative Real-Time PCR Primers				
**Gene**		**Primer Sequence (5' to 3')**	**Annealing Temp (°C).**	**Source**
SNAI1	F	TCGGAAGCCTAACTACAGCGA	60	This study
	R	AGATGAGCATTGGCAGCGAG		
FN1	F	CGGTGGCTGTCAGTCAAAG	60	This study
	R	AAACCTCGGCTTCCTCCATAA		
ACTA2	F	GTGTTGCCCCTGAAGAGCAT	60	This study
	R	GCTGGGACATTGAAAGTCTCA		
CDH2	F	AGCCAACCTTAACTGAGGAGT	60	This study
	R	GGCAAGTTGATTGGAGGGATG		
CDH1	F	ATTTTTCCCTCGACACCCGAT	60	This study
	R	TCCCAGGCGTAGACCAAGA		
VIM	F	GACGCCATCAACACCGAGTT	60	This study
	R	CTTTGTCGTTGGTTAGCTGGT		
ZO-1	F	CAACATACAGTGACGCTTCACA	60	This study
	R	CACTATTGACGTTTCCCCACTC		
BEST1	F	CTGGGCTTCTACGTGACGC	60	This study
	R	TTGCTCGTCCTTGCCTTCG		
CREBBP	F	GAGAGCAAGCAAACGGAGAG	60	Maruotti, 2013
	R	AAGGGAGGCAAACAGGACA		
FBXL12	F	GCCTTGGTCATATCATCAG	60	Maruotti, 2013
	R	TTCTTCATCCGTCCTGTT		
GAPDH	F	TAG CCAAATTCGTTGTCATACC	60	This study
	R	CTGACTTCAACAGCGACACC		
OTX2	F	ACCTTGAACTCCACCTCT	56	Maruotti, 2013
	R	GCTTCTCTTCTCTGACTCTCTTTG		
RPE65	F	TGCGTATGGACTTGGCTTGAATC	56	Maruotti, 2013
	R	TCCTGCTCCTGGGCTCACC		
RLBP1	F	GCTGCTGGAGAATGAGGAAAC	56	Maruotti, 2013
	R	TGGCTGGTGGATGAAGTGG		
BEST1	F	CAGTTCTTCTTCTATGTTG	54	Maruotti, 2013
	R	AATCATCATCATCCTCTC		
MITF	F	TTCACGAGCGTCCTGTATGCAGAT	60	Meyer, 2009
	R	TTGCAAAGCAGGATCCATCAAGCC		
TYR	F	ATTGGGACTGGCGGGATG	56	Maruotti, 2013
	R	GCATAAAGACTGATGGCTGTTG		

ZEB1	F	TTACACCTTTGCATACAGAACCC	60	This study
	R	TTTACGATTACACCCAGACTGC		
TGFB1	F	CTAATGGTGGAAACC CACAACG	60	This study
	R	TATCGCCAGGAATTGTTGCTG		
HEY1	F	CTGAGCTGAGAAGGCTGGT	60	This study
	R	CGAAATCCCAAACTCCGATA		
HES1	F	GTGAAGCACCTCCGGAAC	60	This study
	R	CGTTCATGCACTCGCTGA		
JAG1	F	GAATGGCAACAAAACTTG	60	This study
	R	AGCCTTGTCGGCAAATAG C		
HMGA1	F	AAGCAAAAACAAGGGTGCTG	60	This study
	R	GAGGAAGAGGGCATCTC		
HMGA2	F	GGGCCAGGAGGTAGTTTCTC	60	This study
	R	CCTCGGTGCACCATGTTTGGC		
TWIST1	F	GTCCGCAGTCTTACGAGGAG	60	This study
	R	GCTTGAGGGTCTGAATCTTGCT		
NOTCH1	F	CGCTGACGGAGTACAAGTG	60	This study
	R	GTAGGAGCCGACCTCGTTG		
SNAI2	F	TGTGACAAGGAATATGTGAGCC	60	This study
	R	TGAGCCCTCAGATTTGACCTG		
CTNNB1	F	CATCTACACAGTTTGATGCTGCT	60	This study
	R	GCAGTTTTGTCAGTTCAGGGA		
MICAL1	F	TGCAGAGGAACAACGACAAG	60	This study
	R	AGCCCAGGAGGAAGACAAAT		
MICAL2	F	GGTCAAACCGGAGAGAATGA	60	This study
	R	GCTACGGCTGGAAAAGTTTG		
MICAL3	F	GATGGCTATGCAGGGGTAAA	60	This study
	R	TCCACATTTTGCTCATCCAA		
NRP1	F	ACCCAAGTGAAAAATGCGAATG	60	This study
	R	CACCATGTGTTTCGTAGTCAGA		
RHOA	F	TATCGAGGTGGATGGAAAGC	60	This study
	R	ACTATCAGGGCTGTCGATGG		
NEO1	F	TGGGTCAAAAATGGGGATATGG	60	This study
	R	ACTGAGGATGATGCTGGGAC		
PLXNA3	F	AACCTCGAGAGCAAGAACCA	60	This study
	R	GCTGCTTGATGGCACAGTAA		
HRAS1	F	GTGGGGAACAAGTGTGACCT	60	This study
	R	CATCAGGAGGGTTCAGCTTC		

RAPGDS1	F	TGTGTGGATGCTGGATTGAT	60	This study
	R	AAGAGCTGCATTTTGGCAGT		
RND1	F	TCATTGGCTGCAAGACAGAC	60	This study
	R	GTCCGAAAGATGCTGTGGAT		
ADAM19	F	GAGGGGCGAGAACTGATCCT	60	This study
	R	TGGTTTGAGGGTTACCACTTGA		
ADAMTS1	F	ACGAGTGCGCTACAGATCCT	60	This study
	R	CAGCGTACTTGGGAATCCAT		
BMP7	F	TCGGCACCCATGTTCATGC	60	This study
	R	CTTGGAAAGATCAAACCGGAACT		
EPHA4	F	TTCGCCCTATTTTCGTGTCTC	60	This study
	R	TGGTAGGTTCGGATTGGTGTAT		
EPHB1	F	CTTACGAGGATCCCAACGAA	60	This study
	R	CAAACGCCCCTTGTACACTT		
EPHB2	F	AGTTCGGCCAAATTGTCAAC	60	This study
	R	TCTCCTTGTACTGCCCCATC		
EPHB3	F	GCTACAGCTGGGCTTGTCTT	60	This study
	R	GCAGCTTCTCCGTGTACTCC		
FGFR2	F	GGTGGCTGAAAAACGGGAAG	60	This study
	R	AGATGGGACCACACTTTCCATA		
GNB3	F	ACACCTGGCCAAGATTTACG	60	This study
	R	TAGCTGTCCCACACGATCAG		
GNG11	F	CCTGCCCTTCACATCGAAGAT	60	This study
	R	GGGGTTCTTGTCTTCTGGAAT		
ITGA2	F	CCTGCCCTTCACATCGAAGAT	60	This study
	R	GGGGTTCTTGTCTTCTGGAAT		
ITGA5	F	GCCTGTGGAGTACAAGTCCTT	60	This study
	R	AATTCGGGTGAAGTTATCTGTGG		
NGEF	F	ACCAAGAAGCTCTTCCACGA	60	This study
	R	TGAGAGGACGCCTTTAGCAT		
NRP2	F	CCCTCACTTTGAAATCGAGAAGC	60	This study
	R	CCTTGCCGTTTAGGCTGTAG		
PDGFB	F	TCCCGAGGAGCTTTATGAGA	60	This study
	R	CTCAGCAATGGTCAGGGAAC		
PIK3CD	F	TCGCTCCACCAAGAAGAAGT	60	This study
	R	CGGTCTTAAGCTGGTCCTTG		
PLCH1	F	GGCAATTGTGGCTATGTCCT	60	This study
	R	AGTCTGGAGGTTTGGGGAGT		
PRKCQ	F	CTTCAGAGAAGGTCCGGTTG	60	This study

	R	AACGGAGACTCCCAGGAAAT		
SEMA3A	F	AGACTCACTTGTACGCCTGTG	60	This study
	R	CCCAAGAGTTCGGAAGATAGCAA		
SEMA3D	F	GGAAAGTGCAGACCATCGTT	60	This study
	R	CATCACAGAGTGCCGCTTTA		
SEMA4A	F	AGCCAGCGAGTTTGACTTCTT	60	This study
	R	CGTGGCGGATGACGTTGAA		
SEMA4B	F	GGGTGCAGACGCAGAGAAG	60	This study
	R	AGGTACACATGGGGCTGAAG		
SEMA6D	F	GGCGATCAAGACAACAGGAT	60	This study
	R	AAGAGGGCAACATTGGTTTG		
UNC5D	F	AAGCCCTTCCCGAATCCATC	60	This study
	R	CCACCTGTTGCCTAGTAACATTG		
ABLIM1	F	ACTGCATCTCTCCCTGGCTA	60	This study
	R	TGTTGGTCACCATGAGCATT		
CXCL12	F	AGAGCCAACGTCAAGCATCT	60	This study
	R	CTTTAGCTTCGGGTCAATGC		
CXCR4	F	ACTACACCGAGGAAATGGGCT	60	This study
	R	CCCACAATGCCAGTTAAGAAGA		
EFNA5	F	CGCTACGCTGTCTACTGGAAC	60	This study
	R	ATCGCCAGGAGGAACAGTAG		
EFNB1	F	GAGGCAGACAACACTGTCAAG	60	This study
	R	AGCTTCAGTAGTAGGACCGTC		
EFNB2	F	CTGCTGGATCAACCAGGAAT	60	This study
	R	CTGTTGCCGTCTGTGCTAGA		
NTNG1	F	GCCCTTTGGGTTACGGTGT	60	This study
	R	TCTGTTAGCTCAATGGTTTTGCT		
PLXNB1	F	TCTGCTCAGTGACCTGGTTG	60	This study
	R	GTGTATTTGGCCTTGCCTGT		
RGS3	F	GGCAAGGCAGACAAAATGAT	60	This study
	R	GAACTCAGTGCGAAGGAAGG		
