# Supplementary material for: Transcriptome Landscape of Epithelial to Mesenchymal Transition of Human Stem Cell–Derived RPE
Source: Invest Ophthalmol Vis Sci. 2021 Apr 1;62(4):1. doi: 10.1167/iovs.62.4.1 (PMC8024778; doi:10.1167/iovs.62.4.1)
Supplement: Supplement 4 [file iovs-62-4-1_s004.pdf]

Supplementary Table 2. Antibody Information

Antibody	Source	Dilution
Vimentin (D21H3) Rabbit mAb #5741	Cell Signaling	1/100
ZO-1 (D7D12) Rabbit mAb #8193	Cell Signaling	1/500
Claudin-1 (D5H1D) Rabbit mAb #13255	Cell Signaling	1/100
E-Cadherin (24E10) Rabbit mAb #3195	Cell Signaling	1/200
N-Cadherin (D4R1H) Rabbit mAb #13116	Cell Signaling	1/200
TWIST1 Antibody #46702	Cell Signaling	1/100
RLBP1 Mouse mAb #ab15051	Abcam	1/100
Tyrosinase Rabbit mAb #ab170905	Abcam	1/100
RPE65 Mouse mAb #ab13826	Abcam	1/100
